# Resilience to Hurricanes Is High in Mangrove Blue Carbon Forests

**DOI:** 10.1111/gcb.70124

**Published:** 2025-03-19

**Authors:** David Reed, Selena Chavez, Edward Castañeda‐Moya, Steven F. Oberbauer, Tiffany Troxler, Sparkle Malone

**Affiliations:** ^1^ Yale School of the Environment Yale University New Haven Connecticut USA; ^2^ Department of Earth and Environment Florida International University Miami Florida USA; ^3^ Institute of Environment, Florida International University Miami Florida USA; ^4^ Department of Biological Sciences Florida International University Miami Florida USA; ^5^ Yale Center for Natural Carbon Capture Yale University New Haven Connecticut USA

**Keywords:** carbon cycling, ecosystem disturbance, ecosystem recovery, eddy covariance, mangrove

## Abstract

Mangrove forests are typically considered resilient to natural disturbances, likely caused by the evolutionary adaptation of species‐specific traits. These ecosystems play a vital role in the global carbon cycle and are responsible for an outsized contribution to carbon burial and enhanced sedimentation rates. Using eddy covariance data from two coastal mangrove forests in the Florida Coastal Everglades, we evaluated the impact hurricanes have on mangrove forest structure and function by measuring recovery to pre‐disturbance conditions following Hurricane Wilma in 2005 and Hurricane Irma in 2017. We determined the “recovery debt,” the deficit in ecosystem structure and function following a disturbance, using the leaf area index (LAI) and the net ecosystem exchange (NEE) of carbon dioxide (CO_2_). Calculated as the cumulative deviation from pre‐disturbance conditions, the recovery debt incorporated the recapture of all the carbon lost due to the disturbance. In Everglades mangrove forests, LAI returned to pre‐disturbance levels within a year, and ecosystem respiration and maximum photosynthetic rates took much longer, resulting in an initial recovery debt of 178 g C m^−2^ at the tall forest with limited impacts at the scrub forest. At the landscape scale, the initial recovery debt was 0.40 Mt C, and in most coastal mangrove forests, all lost carbon was recovered within just 4 years. While high‐intensity storms could have prolonged impacts on the structure of subtropical forests, fast canopy recovery suggests these ecosystems will remain strong carbon sinks.

## Graphical abstract

Mangrove forests are an important part of global carbon cycling and are also subject to disturbances from large storms. While initial carbon losses are high following hurricane landfalls, in this work we show that these forests recover to their baseline conditions within 4 years.

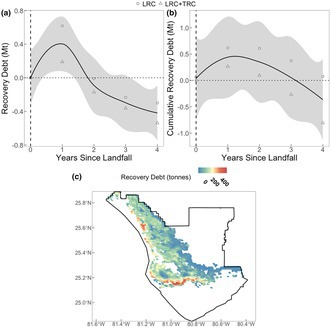

## 1 Introduction

Mangrove forests are strong carbon sinks (Donato et al. 2011; Twilley et al. 1992; Zhu and Yan 2022). In addition to their potential for carbon storage in both vegetation (above‐ and belowground) and the soil, anoxic conditions dampen aerobic respiration (Donato et al. 2011; Lovelock et al. 2017), increasing the capacity for carbon sequestration. Consisting of only 0.5% of the coastal area, mangroves contribute 10%–15% of total coastal sediment carbon storage globally and 10%–11% of total particulate carbon export to the marine environment (Alongi 2014). Thus, mangrove forests are critical to the global carbon cycle (Bouillon et al. 2008; Breithaupt et al. 2012; Breithaupt and Steinmuller 2022; Twilley et al. 1992).

Although important for their capacity to capture and sequester carbon, mangroves are among the most threatened and rapidly disappearing coastal environments worldwide (Valiela et al. 2001). More than 35% of mangrove forests have been degraded or lost over the past two decades due to anthropogenic activities (Giri et al. 2011; Polidoro et al. 2010; Thomas et al. 2017). Occurring in regions with a high frequency of tropical storms and hurricanes, mangrove‐dominated ecosystems exist across subtropical to tropical coastlines, including South Florida, the Gulf of Mexico, and within the Caribbean (Krauss and Osland 2020; Zhang et al. 2019). While the mangrove range has been expanding with warmer temperatures (Osland et al. 2022), storms have changed the structure, functional attributes, and trajectories of forest recovery (Chavez et al. 2023; Danielson et al. 2017; Imbert 2018; Rivera‐Monroy et al. 2019). Sea‐level rise (Cazenave and Cozannet 2014), changes in inundation and sediment relocation (FitzGerald et al. 2008), extreme events (Danielson et al. 2017; Malone et al. 2016), saltwater intrusion (Visschers et al. 2022; Yu et al. 2019), and increased warming (Alongi 2015) have also had significant effects on mangrove ecosystem function. While deforestation is also an ongoing threat to mangrove extent and distribution, multiple international conservation policies and restoration projects have worked to stabilize these forested wetlands and ensure their sustainability (Alongi 2002; Lovelock et al. 2022).

Currently, hurricanes and tropical cyclones are the largest non‐anthropogenic disturbances affecting mangroves worldwide (Sippo et al. 2018). In the North Atlantic Basin, ~15% of the mangrove area (~280,000 ha) has been impacted by hurricanes over the last 25 years (Amaral et al. 2023). A single hurricane can result in high immediate and delayed mortality (10%–45%) in mangrove forests (Barr et al. 2012), and structural damage occurs more frequently in tall forests (> 10 m) due to high wind speeds (Barr et al. 2012; Lagomasino et al. 2021; Radabaugh et al. 2020). Although changes in mangrove structure and function are evident in damaged aboveground biomass, studies suggest that canopy regrowth and recovery of photosynthetic rates (i.e., litterfall productivity) can occur within 1–5 years (Danielson et al. 2017; Lagomasino et al. 2021; Xiong et al. 2022), depending on the severity of the disturbance.

While the magnitude of disturbance impacts is often the focus of research (Amaral et al. 2023; Danielson et al. 2017; Imbert 2018; Lin et al. 2020; Lugo 2008; Rivera‐Monroy et al. 2019), precise evaluation of ecosystem recovery timescales can be challenging due to multiple ecological processes recovering at variable rates. Following Hurricane Wilma in 2005, Barr et al. (2012) examined the net carbon dioxide (CO_2_) exchange (NEE) and the ecosystem respiration (*R*_eco_) of a tall mangrove forest in the Florida Everglades and found different recovery rates for NEE compared to *R*_eco_. The recovery of photosynthetic rates occurred faster, with a near‐complete recovery within 1 year (Barr et al. 2012). However, *R*_eco_ remained elevated after Hurricane Wilma for 4 years due to lingering organic matter decomposition from debris. Hence, the NEE was impacted for many years beyond the recovery of photosynthetic rates (Barr et al. 2012). Similar patterns were shown in dry tropical forests, where there was a ~15% decrease in gross primary production with an increase in soil carbon efflux, soil nitrogen, and litter deposition following a storm (Vargas 2012), as well as a decoupling of respiration from soil temperature while the site was disturbed (Vargas and Allen 2008). Ultimately, carbon exchange rates were controlled by the canopy leaf area, which had cascading impacts on multiple ecosystem processes that were evident on different timescales (Barr et al. 2012; Vargas and Allen 2008).

Ecosystem recovery is complex, and a better understanding of processes in space and time is necessary to measure disturbance‐related feedback to the global climate system. To investigate hurricane effects and subsequent recovery in mangrove forests, we used the remotely sensed leaf area index (LAI) to assess changes in ecosystem structure and NEE to quantify functional impacts on carbon dynamics. We used data from two mangrove sites representing the structural and functional variability observed across the Florida Coastal Everglades landscape (Castañeda‐Moya et al. 2013). We conducted a comprehensive analysis of ecosystem recovery and post‐hurricane ‘recovery debt’ (Moreno‐Mateos et al. 2017) to determine how reductions in ecosystem components co‐occur with changes in NEE, and we evaluated the time required to obtain all the carbon lost because of the disturbance.

This study aims to elucidate structural and functional recovery patterns following tropical storms, focused explicitly on the connection between canopy biomass using LAI and carbon cycling estimates of respiration and maximum photosynthetic rates. We hypothesize that while the recovery of photosynthetic rates will occur quickly, the recovery of respiration will lag. Together, these effects will explain annual deficits and their persistence beyond the recovery of photosynthetic rates. Although regional‐to‐global studies employing remote sensing have underscored the severity of mangrove forest disturbances (Lagomasino et al. 2021), and forest ecosystem trajectories of recovery have been elucidated following specific events (Danielson et al. 2017; Rivera‐Monroy et al. 2019), our understanding of how structural and functional recovery trajectories intersect remains limited.

Given the global importance of mangrove forests for global climate change adaptation and mitigation strategies (Lovelock et al. 2017, 2022; Murdiyarso et al. 2015; Siikamaki et al. 2012) and the growing risk of hurricanes (Balaguru et al. 2023; Holland and Bruyère 2014), there is a pressing need for more refined, long‐term investigations of post‐storm impacts and recovery processes in mangrove‐dominated ecosystems. This is particularly significant in Florida due to the high storm recurrence frequency of ~3 years (Smith 1999), where complex interactions between hurricane legacies (Castaneda‐Moya et al. 2020; Castañeda‐Moya et al. 2010), climate change, and the rapid increase in sea‐level rise during the last decade (Wdowinski et al. 2016) can potentially affect mangrove ecological properties and trajectories of recovery.

## 2 Methods

### 2.1 Study Location and Disturbance History of Everglades National Park

South Florida mangrove forests extend approximately 250,000 ha (Giri and Long 2016). In this subtropical region, precipitation varies between the wet (June to October) and dry (November to May) seasons, with mean annual precipitation being 1430 mm and approximately 60% of the annual precipitation occurring during the wet season (Duever et al. 1994). The mean annual temperature is 23.9°C, with generally slight temperature variation, ranging from a mean monthly minimum of 18.1°C (January) to 29.4°C (August) (Duever et al. 1994).

Significant damage to mangrove forest structure (i.e., defoliation, tree snapping, and uprooting) has been documented from Hurricanes Wilma and Irma (Chavez et al. 2023; Danielson et al. 2017; Lagomasino et al. 2021; Radabaugh et al. 2020; Rivera‐Monroy et al. 2019; Smith et al. 2009; Xiong et al. 2022). Wilma made landfall as a category three storm on the southwestern coast of Florida on October 24, 2005. Approximately 85 km northeast of Marco Island, Florida, Wilma had maximum sustained wind speeds of ~194 km/h^−1^ (Beven et al. 2008). As a category three hurricane, Irma made landfall in the same area on September 10th, 2017, with wind speeds of 180–193 km/h^−1^ (Blake 2018). Both hurricanes produced significant storm surges with maximum water levels up to 1–4 m above the soil surface inside mangrove forests, depositing a thick layer of mineral sediments rich in phosphorus (Castaneda‐Moya et al. 2020; Castañeda‐Moya et al. 2010).

**FIGURE 1:**
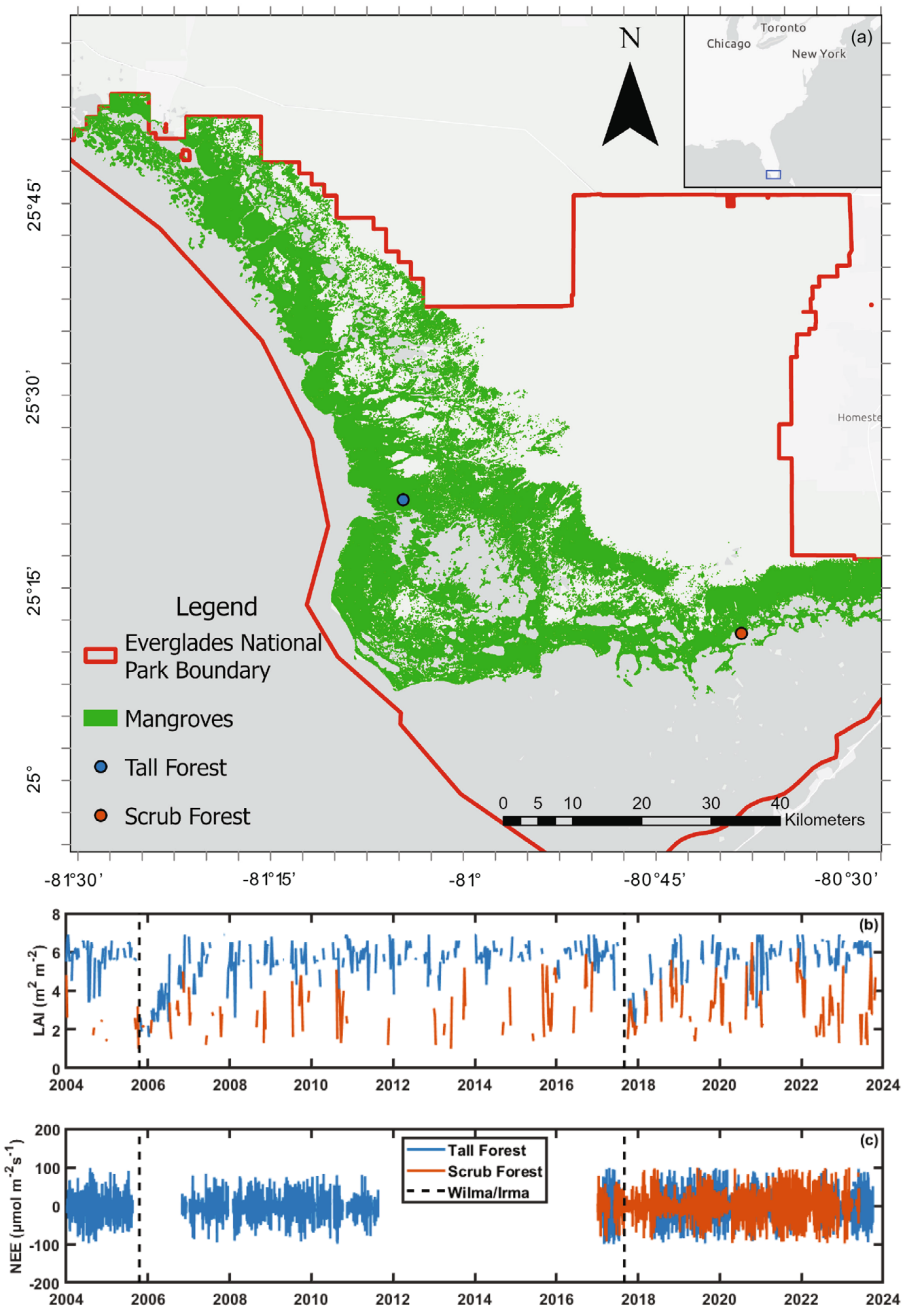
Map of Everglades National Park with park boundaries outlined in red, the spatial extent of mangrove forests in green, and the locations of the mangrove forest and mangrove scrub eddy covariance tower sites (panel a). Time series of leaf area index estimated from MODIS (panel b) and Net Ecosystem Exchange flux of carbon dioxide (panel c, NEE) data from the tall forest mangrove (blue) and scrub mangrove (orange) sites, showing data availability for this study and when Hurricane Wilma impacted the tall forest site in 2005 and when Hurricane Irma impacted both sites in 2017.

Data were collected from two mangrove‐dominated sites in Everglades National Park (Figure 1a), Shark River Slough (SRS6, 25°21′47.9″N 81°04′38.6″ W) and Taylor River Slough (TS/Ph7, 25°11′26.9″N 80°38′20.8″ W). As part of the Florida Coastal Everglades Long‐Term Ecological Research program (Childers et al. 2006, 2019), mangrove sites have been monitored to record forest structure, functional attributes, and soil biogeochemical properties since December 2000 (Castañeda‐Moya et al. 2013). These Everglades sites were representative of the low to mid‐range in canopy height, 1–20 m, and function (100–1170 g Cm^−2^ NEE) observed across mangrove forests globally (Barr et al. 2010; Liu and Lai 2019; Liu et al. 2020; Zhu et al. 2021; Alvarado‐Barrientos et al. 2021; Gnanamoorthy et al. 2020; Wang et al. 2023; Gou et al. 2023; Tian et al. 2024; Jha et al. 2014; Wu et al. 2024).

Located downstream along the Shark River in southwestern Everglades, the tall mangrove forest site (SRS6) was 4 km from the mouth of the estuary. The tall forest site was a mature mangrove forest with an average canopy height of 15–20 m (Barr et al. 2010; Ewe et al. 2006) and was composed of mixed species of *Rhizophora mangle* , *Avicennia germinans* , and *Laguncularia racemosa* . Riverine mangroves in Shark River were tidal‐dominated with well‐defined soil phosphorus (P) fertility and hydroperiod gradients along the estuary. Following patterns in nutrient availability, mangroves were taller and more productive near the coast compared to mid‐ and upstream regions (Castañeda‐Moya et al. 2013; Rivera‐Monroy et al. 2019).

Along Taylor River in the southeastern Everglades, the scrub mangrove (TS/Ph7) was approximately 1.5 km inland from Florida Bay. Mangroves in Taylor River were arranged in small clusters of islands dominated by *R. mangle* scrub mangrove (tree heights < 2.5 m), with the sporadic occurrence of *L. racemosa* and *Conocarpus erectus* in the center of islands, intermixed with low densities of freshwater grasses *Cladium jamaicense* and *Eleocharis cellulose* (Ewe et al. 2006). Mangroves in Taylor River were non‐tidal systems and were permanently flooded (Hogan et al. 2022), which enhanced anoxic conditions and the buildup of porewater sulfides (range: 0.5–2 mM). Coupled with strong soil P limitation (*N*:*p* = 102) in Taylor River, mangrove growth and development were constrained, resulting in low‐stature mature forests (Castañeda‐Moya et al. 2013, 2011; Hogan et al. 2022).

### 2.2 Ecosystem Leaf Area

The LAI is the amount of one‐sided leaf area per horizontal ground area (m^2^ m^−2^), and at spatial scales larger than a single plant, LAI values above ~3 are often considered a full canopy (Carlson and Ripley 1997). Both net and maximum assimilation rates of carbon are well known to depend on LAI at timescales between daily and seasonally (Watson 1958), as higher LAI is proportional to the amount of photosynthetic rate of an individual or ecosystem, depending on the scale of the LAI measurement.

Data was acquired from MODIS Terra and Aqua satellites. With an 8‐day sample period and a 500 m resolution (Myneni et al. 2015), MODIS data was obtained for all mangrove‐dominated ecosystems, as defined by the National Park Service vegetation map (Kevin et al. 2020). The MODIS LAI dataset was screened for cloud cover, aerosol concentration, cloud shadows, and water reflection. The tower pixel had a high degree of water reflection at the scrub mangrove site. Thus, LAI data was acquired from a location close (2.6 km away; 6 pixels) to the scrub mangrove tower. To reduce noise and gaps, the 8‐day MODIS LAI was aggregated by taking the maximum LAI of each 24‐day period (See Text S1 and Figures S1 and S2). To fill gaps in 24‐day maximum LAI, data was linearly interpolated (maximum gap of 2 periods at the forest site, 6 at the scrub site). Data were synchronized to January 1st of each calendar year. Data were accessed via the NASA Earthdata AppEEARS portal (https://appeears.earthdatacloud.nasa.gov/).

### 2.3 Eddy Covariance

The eddy covariance method is a standard approach to measure the net exchange of mass, energy, and momentum between the land surface and the lower atmosphere (Baldocchi 2003). This approach integrates observations from a single location to provide landscape‐level information beyond the measurement footprint (Poe et al. 2020). The net flux of a gas species can be quantified using high‐resolution measurements of turbulent fluctuations of gas concentrations in the surface boundary layer. With a homogeneous land surface below the measurement height and within the measurement footprint, typically on the scale of 1–3 km^2^ upwind of the measurement location, we can assume flux divergence and advection terms are negligible. The flux can be quantified as a sum of the turbulent and storage flux terms of (Equation 1).

(1) $$F_{c}=\bar{\rho_{a}}\bar{w\prime c\prime }\int_{0}^{z}\bar{\rho_{a}}\frac{\partial c}{\partial t}\mathrm{dz}$$

Where $\rho_{a}$ is the air density, $w^{\prime }$ is deviations from the mean vertical wind speed, c is a measured gas species, and $c^{\prime }$ is deviations of the mean gas concentration, measured at height z above the land surface. Here, we defined fluxes as negative for the net uptake of mass, following the standard definition of NEE.

Eddy covariance data for CO_2_ exchange was collected starting in January 2004 at the tall forest (Fuentes et al. 2016) site and in January 2017 at the scrub mangrove site (Malone and Troxler 2024) (Table 1). Two sonic anemometers were used at the tall forest site, first a Gill R3‐50 (2004–2021) and later a Gill WindmasterPro (2022–2023) (Gill Co., Lymington, England). Gas concentrations were measured with a LI‐COR LI‐7500 open‐path infrared gas analyzer (CO_2_ and H_2_O, LI‐COR Inc., Lincoln, Nebraska). At the scrub mangrove site, the sonic anemometer was a CSAT3 (Campbell Scientific, Logan, Utah), and the open‐path infrared gas analyzer was a LI‐COR LI‐7500. Radiation data were measured above the canopy with a Kipp & Zonen CNR4 at the tall forest and a CNR1 at the scrub mangrove site (Kipp & Zonen, Delft, The Netherlands), with observations recorded at 1‐s intervals and averaged to 30‐min timescales.

**TABLE 1:** Site instrumentation information for the tall and scrub forest eddy covariance towers. Measurement height is the distance the sonic anemometer is above ground level, and sensor offset is the distance from the sonic anemometer to the open‐path infrared gas analyzer.

Site	Instrumentation date range (sensor collection frequency)	Canopy height	Measurement height	Sensor offset
Tall forest	2004–2011 (10 Hz) 2017–2023 (20 Hz)	15–20 m	26 m	27 cm horizontal −5 cm vertical
Scrub forest	2017–2023 (20 Hz)	< 2.5 m	4.2 m	16 cm horizontal 0 cm vertical

### 2.4 Eddy Covariance Data Processing

Eddy covariance data from the tall forest site from 2004 to 2012 were processed with a standard approach (Barr et al. 2012), and the data were uploaded to the AmeriFlux database for quality control and archival (Fuentes et al. 2016). Data from both mangrove sites from 2017 to 2023 were processed in EddyPro version 7.0.9 (LI‐COR Inc) following standard flux processing protocols. In the high‐frequency data, spikes larger than 2% were removed, and gaps were filled with linear interpolation (Vickers and Mahrt 1997). Fluxes were adjusted for humidity (Van Dijk et al. 2004) and Webb, Pearman, and Leuning corrections (Webb et al. 1980). High‐pass (Massman 2000) and low‐pass (Moncrieff et al. 2004) filters were also applied. Due to environmental conditions not meeting the cold season air temperature threshold, the surface heat exchange correction for open‐path analyzers under cold conditions (Burba et al. 2008) was not applicable. Quality checks of steady‐state and developed turbulent conditions were based on the correlation coefficient between momentum flux and the NEE. Following Mauder and Foken (2004), data consisting of unsuitable turbulence were removed. Finally, data outside expected ranges, > 100 and < −100 μmol m^−2^ s^−1^ for CO_2_ exchange, were removed. Following flux filtering, when sites were operating, 33.7% of the daytime and 35.7% of nighttime data remained at the tall forest site, and 20.4% of daytime data and 12.0% of nighttime data remained at the scrub mangrove site. Flux tower footprints were estimated with a footprint model with the area approximated as a circle with a radius equal to the mean of the 90% flux contribution distance (Kljun et al. 2015).

The observation system at the tall forest site was offline for some time following severe damage to the tower and instruments by Hurricane Wilma in 2005 and Hurricane Irma in 2017. The tower at the scrub mangrove site had minor impacts from Irma, and data collection restarted 2 months after the storm in November 2017 (Figure 1c). We used ERA5 reanalysis of incoming solar radiation and air temperature data, an hourly product at 0.25°× 0.25 spatial scales, to fill gaps in radiation and temperature at tower sites (Hersbach et al. 2023).

### 2.5 Carbon Exchange Parameters

The eddy covariance method directly measures the NEE of CO_2_, from which uptake and respiration can be partitioned. Flux partitioning methods include the commonly used daytime‐based algorithm of Gilmanov et al. (2003) and Lasslop et al. (2010), which uses a light response curve with a temperature response function. Following a disturbance, it is unclear if *R*_eco_ would follow a typical temperature response. Vargas and Allen (2008) directly showed a decoupling of temperature and respiration that was due to a large increase in the soil‐accessible carbon from defoliation and litterfall, and additional nutrients added to the soil from the storm surge. Gilmanov et al. (2003) and Lasslop et al. (2010) flux partitioning methods would likely underestimate respiration following a disturbance and then hence overestimate uptake rates, as uptake was derived from the difference between the measured net flux and the temperature response‐derived ecosystem respiration.

We explored two methods of estimating ecosystem‐level carbon exchange parameters. First, we used light response curves (LRC) to quantify changes in daytime carbon exchange parameters, avoiding assumptions of constant parameters during the disturbance recovery period (Reichstein et al. 2005). This approach holds *R*_eco_ constant, which assumes the daytime *R*_eco_ is similar to nighttime rates. Daytime, 30‐min NEE was used to parametrize LRC in 24‐day periods (Text S1) starting January 1st of each year to fit a Michaelis–Menten type light response curve following (Equation 2):

(2) $$\mathrm{NEE}=\frac{\mathrm{QY}\cdot A_{\max}\cdot R_{g}\;}{\mathrm{QY}\cdot R_{g}+A_{\max}}+R_{\mathrm{eco}}$$

where incoming solar radiation (*R*_g_) was used to estimate the initial slope of the light curve (quantum yield; QY), *A*_max_ is the asymptotic value at high light values, and ecosystem respiration (*R*_eco_) is the intercept term. Daytime (*R*_g_ > 50 W m^−2^) data was used to fit (Equation 2). We used the lsqcurvefit function in MATLAB, a nonlinear least‐squares solver algorithm that iteratively changed the parameters to minimize the square error between the data and a parameterized function. Initial conditions (and ranges) were set to QY = −0.01 (−0.4 to 0.0), *A*_max_ = 10 (−60 to −0.01), and *R*_eco_ = 1 (0–100). Curves were fit for 24‐day periods with at least 40% of the data available. The *R*_eco_ estimated with this LRC is hereafter referred to as LRC *R*_eco_.

The second approach to estimating NEE and carbon exchange parameters incorporated Equation (2) for daytime data and used temperature response curves (TRC) at night. TRC were fit with nighttime NEE (*R*_g_ < 50 W m^−2^) following Reichstein et al. (2005), where ecosystem respiration was modeled as (Equation 3):

(3) $$R_{\mathrm{eco}}={\mathrm{rb}}^{E0\left(\left(\frac{1}{\text{Tref}-T0}\right)-\left(\frac{1}{\text{Tair}-T0}\right)\right)}$$

where rb is the base respiration at the reference temperature, *Tref* is set to 15°C, E0 is the temperature sensitivity, *Tair* is the air temperature, and the *T0* parameter is kept at a constant −46.02°C. Curve fitting was performed similarly to the light response curve above, with at least 40% of the data required for each 24‐day period and with initial conditions (and ranges) set to rb = 100 (0–1000) and E0 = mean of nighttime NEE values (50–400). Following the definition of NEE where net carbon uptake is negative, both QY and *A*_max_ were similarly defined as negative, while *R*_eco_ was positive. The *R*_eco_ estimated with the TRC is hereafter referred to as TRC *R*_eco_.

Barr et al. (2012) used LRC to understand patterns in productivity and TRC to estimate ecosystem respiration, an approach used to understand NEE in Everglades tower sites (Jimenez et al. 2012; Malone et al. 2014; Malone et al. 2015; Malone et al. 2016; Zhao et al. 2019; Malone et al. 2021 ). Due to complicated variability in the environmental factors (e.g., hydroperiod, tidal influence) that drive net fluxes in mangrove forests, it may be difficult to parse out the disturbance recovery timeline using daytime fluxes alone.

### 2.6 Landscape Modeling of NEE

While carbon exchange rates can be directly measured at the site level, the development of landscape‐level estimates of hurricane recovery will require ecosystem properties that can be measured spatially and used to scale carbon exchange parameters. Here, we examined the relationship between LRC parameters (Equation 2) and LAI at the site level. We defined a general nonlinear mixed effect model for light and temperature response curve parameters for the time series of length *i* and with *j* number of responses:

(4) $$y_{i}=\exp\;\left(\alpha_{j}∙{\mathrm{LAI}}_{i}\right)+\alpha_{j}∙\text{Time}+\alpha_{j}∙\text{Structure}+\alpha_{j}∙\text{Tair}+\alpha_{j}$$

where LAI is the leaf area index for a location, Time is the number of days since hurricane landfall and is a linearly increasing factor starting at the hurricane landfall date. Structure is an indicator for tall or short‐stature forest defined as either LAI ≤ 4 or LAI > 4, Tair is the mean daily air temperature and $\alpha_{j}$ are response weights. The model was fit for 24‐day periods, using a least‐squares approach, with an initial response weight of 1 for all variables.

After estimating landscape carbon exchange parameters using Equation (4), we modeled NEE using interpolated LAI and the ERA5 radiation product for the light‐based NEE (LRC) and the ERA5 air temperature product for temperature‐based NEE (TRC) at night. This approach allowed for the continuous estimation of NEE when the flux towers were offline directly following hurricane landfall. At the site and landscape scale, all spatial variation in NEE was due to differences in LAI and their resulting impact on carbon exchange parameters (Equation 4). This approach was validated at the tower sites and then used across mangrove‐dominated ecosystems in the Florida Everglades. We used two methods of estimating NEE: LRC and LRC + TRC, where daytime NEE was estimated with the LRC and nighttime NEE was estimated with TRC.

### 2.7 Hurricane Impacts

Since natural ecosystems are dynamic in time, the best method to quantify disturbance impacts and duration is an open question. Here, we used a modified approach following a relative non‐stationary test, originally developed for atmospheric wind speed measurements (Equation 5), (Vickers and Mahrt 1997), to understand when changes were noted in the time series of carbon exchange parameters (Equations 2 and 3).

(5) $${\mathrm{RN}}_{x}=\frac{\mathrm{dx}}{\left\langle x\right\rangle }$$

where the relative non‐stationarity (*RN*) of a given parameter was calculated using linear regression to estimate the difference (dx) in the parameter over a three‐year period, which was normalized by the mean over that time period $\left\langle x\right\rangle$. With this approach, a value of 1 denoted a stable variable (see Text S2 for additional information). Relative non‐stationarity had been used on variable length time series (Pan and Patton 2017; Večenaj and De Wekker 2014; Vickers et al. 2010) and assumed no long‐term trend in the observations. Annual frequencies in the data (e.g., the annual cycle of *T*_air_) were accounted for within the linear regression slope computation. Impact thresholds of 95th, 90th, and 85th percentiles from the entire measurement record were calculated to estimate when deviations from stationarity and when the return to stationary occurred. The impact duration timescales were defined as the last date within a given impact threshold and reported as the number of days the impact lasted.

### 2.8 Ecosystem Recovery

We measure the post‐storm reduction in ecosystem structure and function during the recovery process, termed the ‘recovery debt’ (Moreno‐Mateos et al. 2017). Defining a reference level, we measured the annual deficit to understand differences in the magnitude and recovery rates of processes and ecosystem components that influence carbon dynamics. Following the disturbance and recovery debt framework of Moreno‐Mateos et al. (2017), the recovery debt was expressed as a reduction in a given ecosystem attribute during the recovery period (Equation 6):

(6) $$\text{Recovery Debt}=\sum \bar{{\mathrm{NEE}}_{\mathrm{pre}}}-{\mathrm{NEE}}_{\text{post}}$$

where the recovery debt is the sum of the difference between the mean condition prior to (NEE_pre_) or during a non‐disturbed period and the condition (NEE_post_) following the disturbance.

With relatively little variability observed in the carbon exchange parameters for undisturbed mangrove sites (Figure S14), NEE_pre_ was defined as the mean of 1 year before landfall. The recovery debt was calculated for NEE at the site level as the annual sum of both LRC NEE_post_ and LRC + TRC NEE_post_, using a four‐year recovery timescale following landfall based on Barr et al. (2012). Using the landscape‐level NEE (LRC and LRC + TRC), the recovery debt of NEE was also estimated for each mangrove‐dominated MODIS pixel within Everglades National Park. We determined both the recovery of ecosystem function and the recovery of all lost carbon by calculating the cumulative sum of the recovery debt of NEE over time at the site and landscapelevels. We estimate the mean debt of LRC NEE and LRC + TRC NEE and report the standard error to show the variation between approaches. To understand patterns in annual recovery debt by years since landfall, we used locally estimated scatterplot smoothing (LOESS), a nonparametric regression method, to fit a smooth curve to data points (Jacoby 2000). This approach was useful in exploratory data analysis, where the goal was to visualize trends without making assumptions about the data.

### 2.9 Statistical Methods

The mean LAI and carbon exchange parameters were calculated for all years and on a time since disturbance basis based on the landfall dates of Hurricanes Wilma and Irma in 2005 and 2017, respectively. Probability density functions of fluxes and model parameters were estimated via a kernel distribution fit using a normal kernel function, and histograms were normalized by relative percentage. The assumption of normality was tested with a one‐sample Kolmogorov–Smirnov test, with all model parameters found to be normally distributed at the 5% significance level. Differences in distributions were conducted using the nonparametric two‐sample Kolmogorov–Smirnov test, which did not assume a normal distribution of the dataset, with all tests using the *p*‐value threshold of 0.05. Data analysis was performed in MATLAB R2023b for site‐level analysis and in R for landscape‐level calculations.

## 3 Results

### 3.1 Variation in LAI and Carbon Exchange Parameters

**FIGURE 2:**
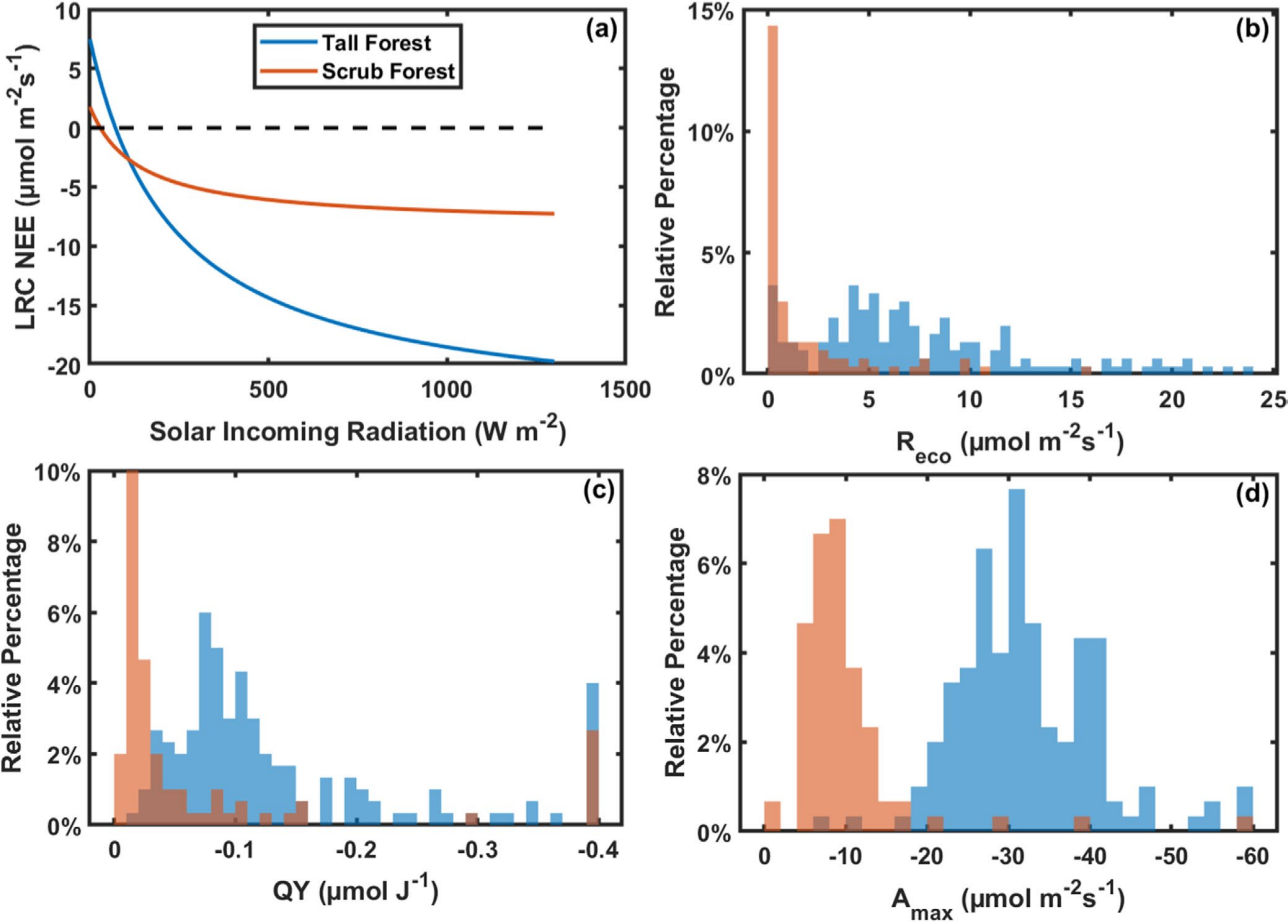
Light response curves (LRC) showing the net ecosystem exchange of CO_2_ (NEE) as a function of incoming solar radiation at the tall forest mangrove tower (blue) and scrub mangrove tower (orange) based on average parameters over the entire study period (a; forest: 2004–2011, 2017–2023, scrub:2016–2023). Distributions for carbon exchange parameters: Ecosystem light response curve‐based respiration (LRC *R*_eco_; b), quantum yield (c), and maximum assimilation of carbon (*A*_max_; d).

**FIGURE 3:**
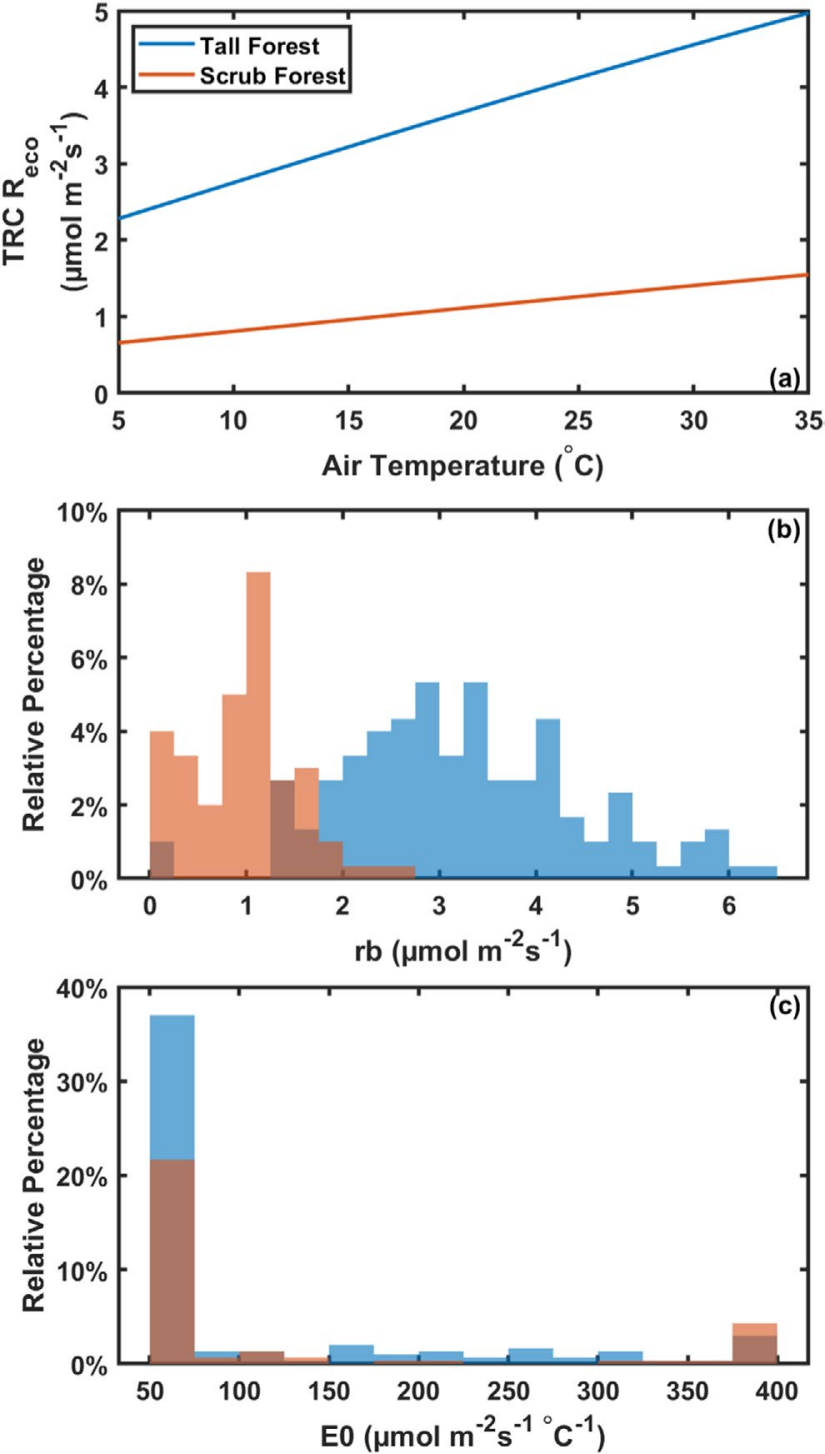
Temperature response curves (TRC) showing temperature response curve‐based respiration (TRC *R*_eco_) as a function of air temperature at the tall forest mangrove tower (blue) and scrub mangrove tower (orange) based on average parameters over the entire study period (a; forest: 2004–2011, 2017–2023, scrub: 2016–2023). Distributions for carbon exchange parameters: Base respiration rate (rb; b) and temperature sensitivity parameter (E0; c).

We measured the variation in LAI within two mangrove forests, focusing on differences that influence carbon exchange (Figure 1b). The tall mangrove forest site had a higher average LAI (5.55 ± 0.03 m^2^ m^−2^) that typically ranged between 4 and 7 m^2^ m^−2^. The LAI at the scrub mangrove site ranged from 0.3–4 m^2^ m^−2^, and the average LAI was 2.87 ± 0.04 m^2^ m^−2^ (Table 2). Between the tall and scrub mangrove sites, there were notable differences in carbon exchange parameters averaged across the entire study period (Figure 2, Table 2). In the tall mangrove forest site, there was a higher average *A*_max_ (−32.14 ± 0.50 μmol m^−2^ s^−1^) and higher average LRC *R*_eco_ (7.49 ± 0.32 μmol m^−2^ s^−1^), relative to the scrub mangroves where the average *A*_max_ was 9.97 ± 0.44 μmol m^−2^ s^−1^ and the average LRC *R*_eco_ was 1.76 ± 0.17 μmol m^−2^ s^−1^ (Table 2). The taller, more developed, dense, and closed canopy in the tall forest resulted in a higher average QY (−0.14 ± 0.006 μmol J^−1^) relative to the sparse canopy of the scrub mangrove where the average QY was −0.07 ± 0.007 μmolJ^−1^ (Table 2). Distributions of parameters show consistently higher parameter values for the tall mangrove forest site compared to the scrub mangrove site (Figure 2b–d). There were differences in rb rates between sites, leading to higher TRC *R*_eco_ rates at the forest site (Figure 3a). The average rb rate was 3.22 ± 0.07 μmol m^−2^ s^−1^ at the tall forest site and 0.96 ± 0.03 μmol m^−2^ s^−1^ at the scrub site (Figure 3b, Table 2). The respiration sensitivity to the temperature parameter of E0 was similar between sites, 107.43 ± 6.04 μmol m^−2^ s^−1^ at the tall forest and 118.36 ± 7.43 μmolm^−2^ s^−1^ at the scrub site (Figure 3c, Table 2).

**TABLE 2:** Mean values of leaf area index (LAI), light response curve parameters of ecosystem respiration (LRC *R*_eco_), quantum yield, and maximum assimilation of carbon (*A*_max_), and temperature response curve parameters of base respiration (rb) and respiration temperature sensitivity (E0), as measured by eddy covariance (EC) at both tall forest and scrub mangrove sites in Everglades National Park. Impact timescale for LAI, light response curve parameters, and mean 30‐min flux rates for net ecosystem exchange (NEE) using return from non‐stationarity test results using 24‐day aggregated data.

Site			LAI [m^2^ m^−2^]	LRC *R*_eco_ [μmol s^−1^ m^−2^]	*A*_max_ [μmol s^−1^ m^−2^]	QY [μmol J^−1^]	rb [μmol s^−1^ m^−2^]	E0 [μmol s^−1^ m^−2^°C^−1^]	EC NEE [μmol s^−1^ m^−2^]
Tall mangrove forest			5.55	7.49	−32.14	−0.1367	3.22	107.43	—
Scrub mangrove			2.87	1.76	−9.97	−0.0738	0.96	118.36	—
	**Impact Threshold**	**Hurricane**	**LAI [days]**	**LRC *R*_eco_ [days]**	***A*_max_ [days]**	**QY [days]**	**rb [days]**	**E0 [days]**	**EC NEE [days]**
Tall mangrove forest	85 Percentile	Wilma	198	—	—	78	—	78	—
		Irma	124	460	729	—	—	148	220
	90 Percentile	Wilma	126	—	—	49	—	—	—
		Irma	95	412	729	—	—	47	148
	95 Percentile	Wilma	78	—	—	25	—	—	—
		Irma	23	—	633	—	—	47	47
Scrub mangrove	85 Percentile	Irma	—	47	—	—	23	268	878
	90 Percentile	Irma	—	23	—	—	—	244	825
	95 Percentile	Irma	—	—	—	—	—	196	777

Overall, respiration estimated from light and TRC was generally in good agreement at the mangrove scrub sites (average *R*_eco_ rates < 5% of each other). However, LRC *R*_eco_ was 78% larger in magnitude at the mangrove forest site compared to the TRC *R*_eco_ (Table S1). TRC‐based *R*_eco_ was less dynamic in time than LRC *R*_eco_ for both sites (Figures S4 and S5).

**FIGURE 4:**
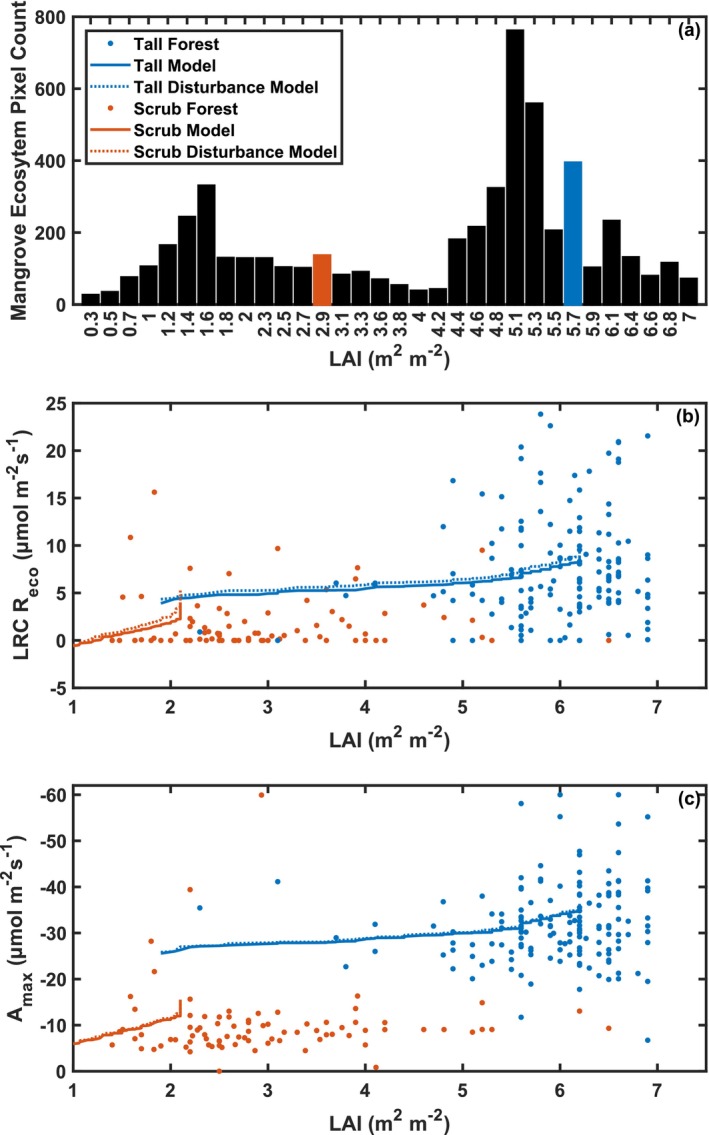
LAI distribution across mangrove forest areas within the Everglades National Park (a). Modeled carbon exchange parameters: Ecosystem light response curve‐based respiration (LRC *R*_eco_; b) and maximum carbon assimilation (*A*_max_; c) as a function of LAI. Models for *R*_eco_ and *A*_max_ are shown (solid line) along with models for disturbance years (dotted line) for data from the tall forest (blue) and scrub (orange) mangrove.

At the landscape scale, a bimodal distribution of LAI across the tall forest and scrub mangroves (Figure 4a) was observed in similar ranges at both tower site locations. Carbon exchange parameters of LRC *R*_eco_ and *A*_max_ scaled exponentially with LAI (Figure 4, Table 3) across the tall forest and scrub mangrove sites. They were used to estimate carbon exchange parameters from MODIS LAI. The LRC *R*_eco_ and *A*_max_ were modeled with a mixed‐effect model (Equation 4), while QY was best estimated with *T*_air_ and the mean of each structure type (Table 3). Both LAI and *T*_air_ were positively correlated with modeled LRC *R*_eco_ and *A*_max_, indicating that increases in either variable resulted in higher modeled estimates. Modeled LRC *R*_eco_ increased with time since disturbance, while modeled *A*_max_ decreased with time. Both LRC *R*_eco_ and *A*_max_ were larger in magnitude at the more structurally developed mangrove site. The coefficients of determination were 0.66, 0.08, and 0.31 for *A*_max_, QY, and LRC *R*_eco_, respectively (Table 3, Figure S2). For TRC, rb was modeled with a mixed‐effect model that increased with time since disturbance and was larger at the forest site, with a coefficient of determination of 0.55 (Table 3).

**TABLE 3:** Nonlinear mixed‐effect model estimates for the relationship between light response curve carbon exchange parameters of ecosystem respiration (LRC *R*_eco_), quantum yield (QY), maximum assimilation of CO_2_ (*A*_max_), and the temperature response curve carbon exchange parameter of base respiration rate (rb) as a function of leaf area index (LAI), time since disturbance (number of 24‐day periods since hurricane landfall for 4 years following landfall), ecosystem structure (tall vs. scrub mangrove), and mean air temperature for each 24‐day period.

	LRC *R*_eco_ model		QY model		*A*_max_ model		rb Model	
	Estimate	95% CI	Estimate	95% CI	Estimate	95% CI	Estimate	95% CI
Exp(LAI)	0.25	(0.17, 0.34)	—	—	0.33	(0.24, 0.41)	—	—
Time Since Disturbance	0.03	(0.003, 0.065)	—	—	−0.01	(−0.07, 0.04)	0.01	(0.005, 0.019)
Structure	−3.91	(−6.02, −1.78)	−0.067	(−0.099, −0.035)	−19.38	(−22.90, −15.85)	−2.27	(−2.59, −1.96)
Air Temperature	0.29	(0.10, 0.47)	0.002	(−0.002, 0.006)	0.63	(0.31, 0.94)	—	—
Intercept	−4.82	(−10.41, 0.77)	0.09	(−0.019, 0.195)	10.15	(0.92, 19.38)	3.01	(2.77, 3.20)
*R* ^2^	0.31		0.08		0.66		0.55	

### 3.2 Hurricane Impacts

**FIGURE 5:**
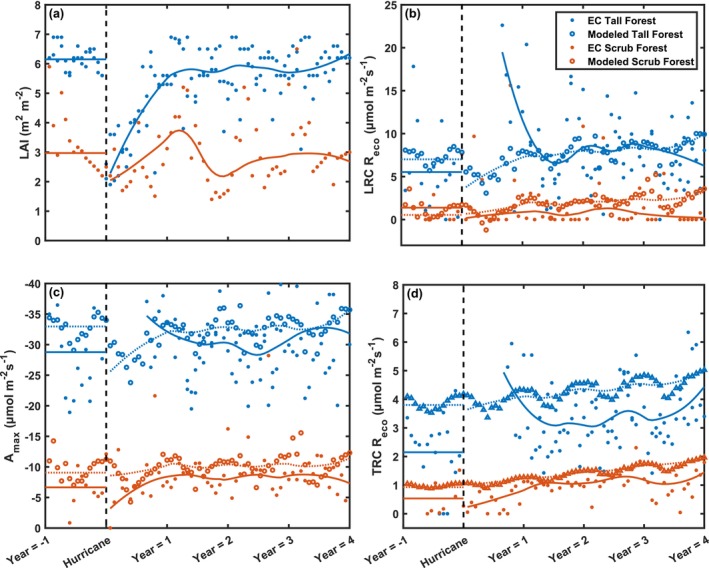
Mean values for LAI (a) and carbon exchange parameters: Light response curve‐based ecosystem respiration (LRC *R*_eco_) (b), *A*_max_ (c), and temperature response curve‐based ecosystem respiration (TRC *R*_eco_) (d) based on time since hurricane landfall. Carbon exchange parameters are shown both for eddy covariance measurements (filled circles, solid lines) and modeled from light (open circles, dotted lines) and temperature response curves (open triangles, dotted lines). Mean values from non‐disturbance impacted times are shown as a horizontal line 1 year before landfall, and smoothed values post‐landfall are shown highlighted with a 2nd‐degree polynomial local regression.

Hurricane impacts on carbon exchange parameters were evident for the tall forest and scrub mangroves. Following the hurricane's landfall, there was a persistent reduction in LAI at the tall mangrove forest but no changes in mean LAI at the scrub mangrove (Figure 5a, Figure S7). The LAI reduction at the tall forest site lasted an average of 111 days after both hurricanes impacted the mangrove area, using the 90th percentile threshold (Table 2).

Based on the mean flux rates at the tower sites, hurricanes dampened carbon exchange parameters immediately after the storm (Figure 5). Small changes in LRC *R*_eco_ occurred immediately following Hurricane Wilma at the tall forest site. However, following Hurricane Irma, reductions in LRC *R*_eco_ rates persisted for 412 days (Table 2, Figure S8) before they began to rise at the tall mangrove site. At the scrub mangrove site, LRC *R*_eco_ was only reduced for 23 days following Hurricane Irma (Table 2, Figure S8). Like LRC *R*_eco_, QY was reduced for 49 days at the tall forest site following Hurricane Wilma, and there was no evidence of any impact at the scrub mangrove site (Figure S9). For *A*_max_, reductions at the tall mangrove site were not detected following Hurricane Wilma, but reductions in *A*_max_ persisted for 729 days following Hurricane Irma (Table 2, Figure S10). While declines in carbon exchange parameters were evident in years 1–2 following the disturbance, changes in LRC *R*_eco_ relative to *A*_max_ led to the eventual stabilization of mean 30‐min NEE rates within a few years.

**FIGURE 6:**
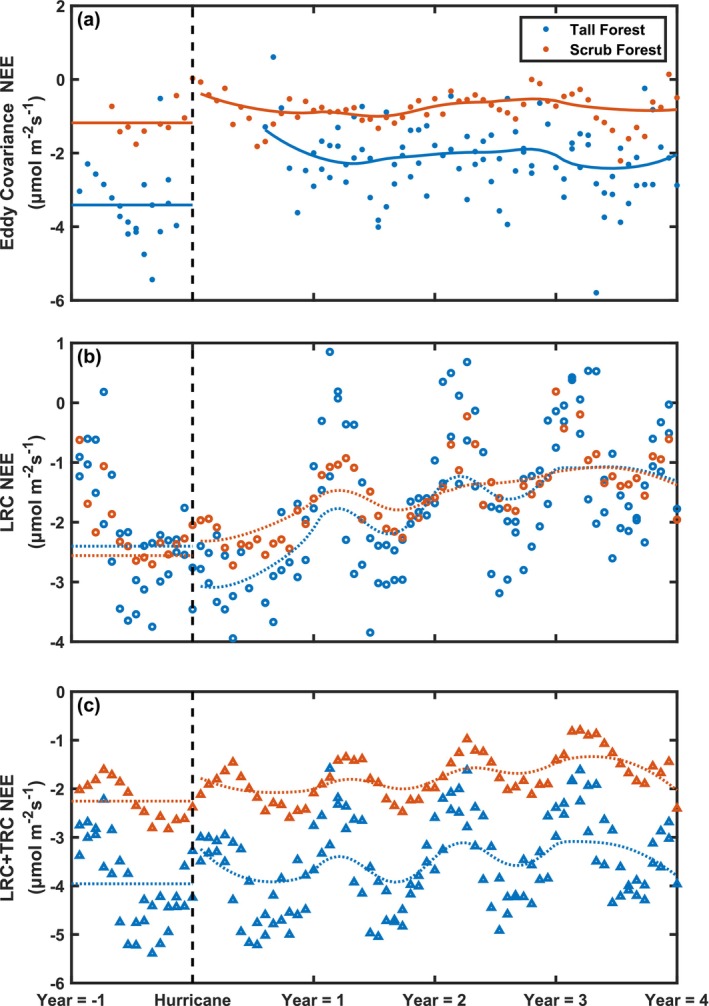
Mean 30‐min flux rates for Net Ecosystem Exchange (NEE) based on eddy covariance measurements (a, filled circles), light response curves (b, open circles), and integrated light and temperature response curves (c, open triangles) based on time since hurricane landfall. 24‐day average data is shown for tall forest (blue) and scrub (orange) mangrove sites. Mean undisturbed condition is indicated with a horizontal line 1 year before landfall, and smoothed NEE values post‐landfall are summarized with a 2nd‐degree polynomial local regression for both eddy covariance NEE (a solid line) and modeled NEE (b and c, dotted line).

Overall, models show that both LRC *R*_eco_ and *A*_max_ were responsive to LAI, so they recovered at similar rates. At the same time, QY, rb, and E0 did not show disturbance impacts. For both sites, measured and modeled *R*_eco_ generally agreed. However, there was an important difference between the modeled LRC and TRC *R*_eco_ and the measured *R*_eco_ 1 year after the disturbance (Figure 5). Accounting for the reduction in LAI and air temperature at the mangrove sites, models estimate an increase in *R*_eco_, though smaller in magnitude than the observations at the tower site. Still, both observations and models indicate that the rates of *R*_eco_ were larger at the tall forest site (Figures 5b and 4d), and there was no reduction in uptake rates at the scrub mangrove site following Irma (Figure S10). Modeled *A*_max_ at the tall forest site decreased as a function of LAI (Figure 5c) following hurricane landfall. The models and data captured fast returns to pre‐disturbance rates. Examining mean half‐hourly carbon exchange rates at the site level, we show that the mean half‐hourly NEE rate increased (more negative) directly following hurricane impact for light‐based models (Figure 6b). At the scrub mangrove site, the mean daily LRC NEE flux rate range remained unchanged following landfall. The seasonal temperature pattern strongly influenced LRC + TRC NEE models, and little hurricane response was noted (Figure 6c).

### 3.3 Ecosystem Recovery

**FIGURE 7:**
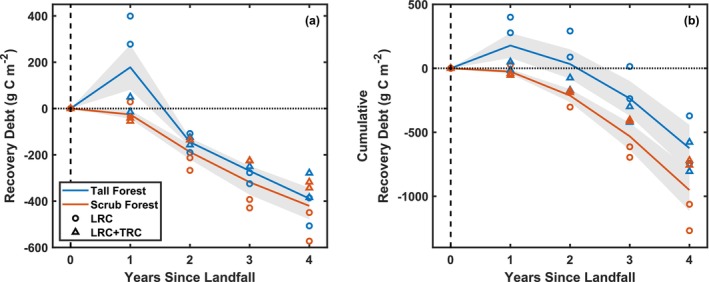
The annual recovery debt (a) and cumulative yearly recovery debt (b) for mangrove tall forest (blue) and scrub forest (orange) for Hurricanes Wilma and Irma by year since landfall. We used two methods of estimating the net ecosystem exchange rate (NEE): light response curves (LRC) and integrated LRC and temperature response curves (LRC + TRC). Negative values indicate net uptake or enhanced carbon capture, and positive values show carbon debt.

There was a maximum carbon recovery debt of 224 ± 223 g Cm^−2^ (Hurricane Wilma) and 132 ± 207 g Cm^−2^ (Hurricane Irma) at the tall forest site in year 1, and we observed that the tall forest site recovered within 2–3 years (Figure 7a). While there was generally little annual recovery debt noted at the scrub site for the first year, the scrub forest continued accumulating carbon 1 year following landfall (Figure 7).

**FIGURE 8:**
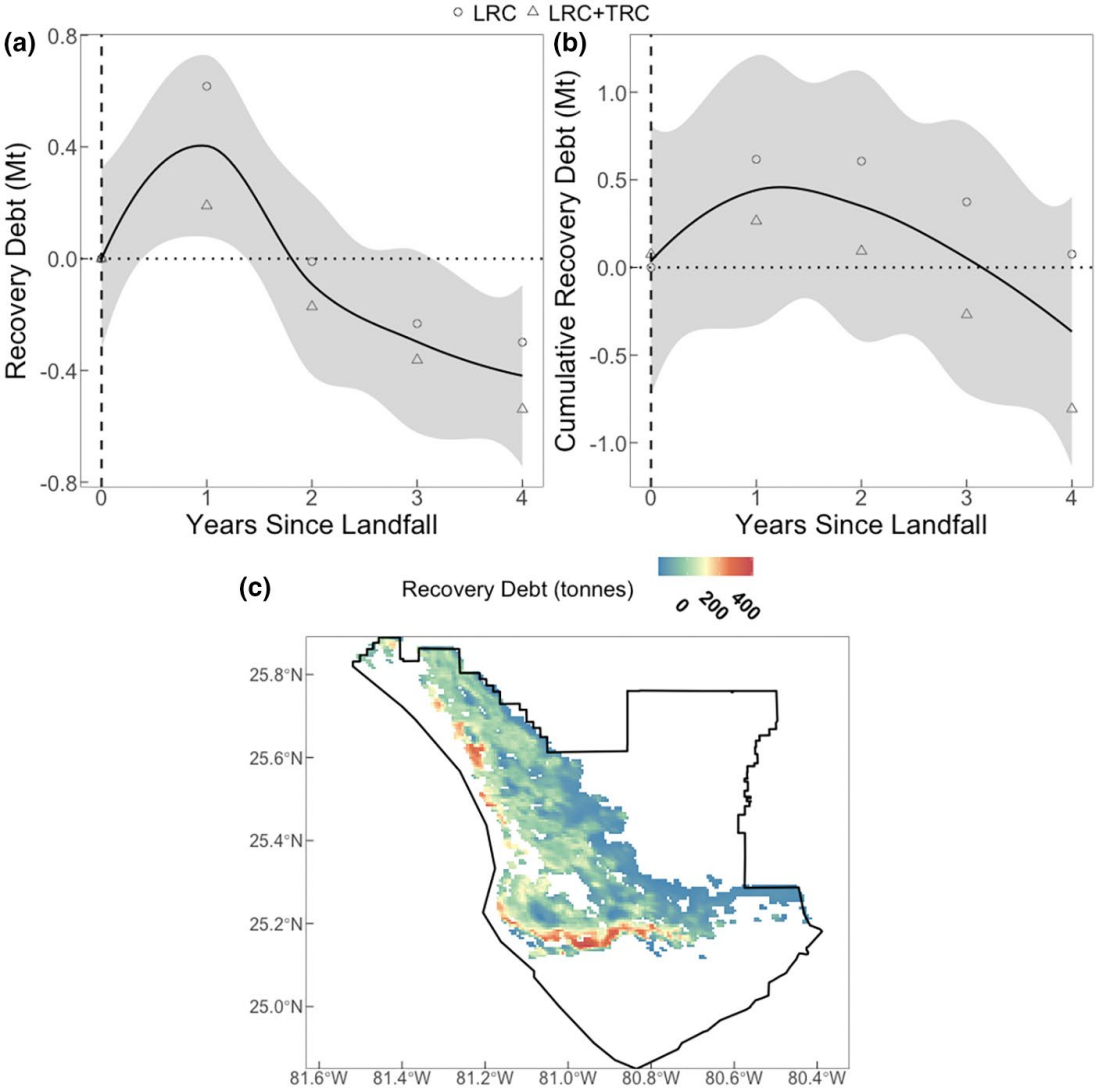
The mean annual landscape debt (a) and the cumulative landscape recovery debt (b) for mangrove net ecosystem exchange of CO_2_ (NEE) in megatons of C for Everglades National Park. The mean annual landscape recovery debt for NEE at 4 years after a hurricane in tonnes of C (c). We used two methods of estimating NEE: light response curves (LRC) and integrated LRC and temperature response curves (LRC + TRC). Negative values indicate net uptake or enhanced carbon capture, and positive values show carbon debt.

At the landscape scale, substantial losses in carbon were largely recovered and surpassed within 4 years following the disturbance (Figure 8). Immediately post‐disturbance (year 1), the average debt was highest (0.40 ± 0.21 Mt. C), and the landscape uptake quickly recovered the capacity to capture carbon so that at 2 years following disturbance, the NEE was restored. After 4 years, all lost carbon had been recaptured. Although most of the mangrove landscape showed an enhancement of carbon capture following recovery (69%), there were still mangrove areas along the coastline that showed a debt at 4 years. However, enhanced carbon capture in other areas offset these localized debts, with a net landscape total of −0.42 ± 0.12 Mt. C in year 4.

## 4 Discussion

### 4.1 Disturbance and Recovery Timescales

Recovery is a dynamic process, and since hurricanes alter multiple facets of ecosystems (Castaneda‐Moya et al. 2020; Danielson et al. 2017; Peneva‐Reed et al. 2021; Rivera‐Monroy et al. 2010), the subsequent post‐storm impact and recovery processes occur at various timescales. In Everglades mangrove forests, rb, QY, and mean average 30‐min NEE rates did not exhibit hurricane impacts. Following the disturbance, LAI, *A*_max_, *R*_eco_, and annual NEE showed impacts that lasted anywhere from < 1 year to +3 years. With additional observations from Hurricane Irma, we found that impacts to *A*_max_, *R*_eco_, and total annual NEE did not last as long as the recovery estimates shown in Barr et al. (2012). Barr et al. (2012) suggest that NEE rates in the tall forest returned to pre‐storm conditions in ~4 years following Hurricane Wilma. Integrating data from multiple storms and sites, we show that annual NEE returned within 3 years and carbon lost through vertical fluxes was recaptured within 4 years at sites showing post‐storm impacts. Storms can cause massive canopy defoliation (Danielson et al. 2017), leading to tree mortality, either directly from the storm's wind damage or through delayed death due to bark and wood damage. We observed that the hurricane‐related decline in LAI in the tall forest led to changes in *A*_max_ and, in turn, *R*_eco_. Enhanced *R*_eco_ was maintained for years, as downed coarse woody debris was broken down (Schmid et al. 2016), influencing the time required for an ecosystem to return to the pre‐disturbance NEE rates (Barr et al. 2012).

While storms can significantly damage ecosystems, they also increase resources essential in subtropical landscapes. High wind speeds caused large storm surges, bringing in additional sediment and nutrients into the mangrove forest floor (Castaneda‐Moya et al. 2020) that could enhance both *R*_eco_ and *A*_max_. Nearly 4 cm of sediments rich in P were deposited at the tall forest site following Hurricane Irma in 2017, with the average soil accretion rate during the disturbance being 6.7 to 14.4 times greater than the long‐term average annual accretion rate (Castaneda‐Moya et al. 2020). A similar resource deposition after Hurricane Wilma also led to a doubling of soil P concentrations, which persisted for 5 years (Davis et al. 2018). Vargas and Allen (2008) showed that these immediate hurricane effects impacted soil respiration (+18%) that persisted for a year, with 67%–70% of this respiration coming from the upper soil layer (2–8 cm of depth). Also highlighting the changes in ecosystem‐level processes, Vargas and Allen (2008) showed a decoupling of soil respiration and temperature, with higher respiration values observed at night, suggesting additional biogeochemical controls on soil respiration besides soil temperature and moisture during disturbance recovery periods.

### 4.2 Understanding Ecosystem Resilience

Overall, mangrove forests are resilient to disturbance (Alongi 2008; Amaral et al. 2023) in the face of projected increases in hurricane frequency (Balaguru et al. 2023). Here, we focused on the canopy leaf area as a proxy for the photosynthetic biomass of the system and showed that impacts to LAI can last less than a year and are relatively shorter than impacts to *A*_max_ and annual NEE. Using canopy height and remote sensing tools, Xiong et al. (2022) found that canopies recover within 2.5 years for the tallest (> 20 m) mangrove forests, and shorter canopies were more resilient with less damage and faster recovery times, while Danielson et al. (2017) found recovery of mangrove resiliency within < 5 years. While canopy damage was correlated to canopy height, with higher canopies more likely to be damaged, rates of post‐hurricane recovery appear to be globally consistent (Peereman et al. 2022). In the coming decades, tropical storms will strengthen and become more frequent (Balaguru et al. 2023). In the Atlantic Basin, this would result in more landfalls along the Gulf Coast and the lower Atlantic East Coast (south of 40° N) (Balaguru et al. 2023). Ultimately, future storms will likely impact previously unaffected areas, both inland mangroves during stronger storms and poleward mangroves from hurricane range expansion. Coupled with other stressors such as drought, the total mangrove canopy damage from tropical storms could increase globally (Peereman et al. 2022).

A central component of ecosystem resiliency and recovery from disturbance is that hurricanes can act as fertilizing events (Castaneda‐Moya et al. 2020; Davis et al. 2018). An increase of between 49% and 98% in soil P levels was observed in Everglades mangrove forests and helped to stimulate rapid forest recovery following hurricane disturbances (Castaneda‐Moya et al. 2020; Davis et al. 2018). These soil nutrient inputs lead to significant increases in soil porewater and leaf litter P concentrations, which facilitate rapid forest regrowth and recovery and stimulate soil biomass accumulation (Castaneda‐Moya et al. 2020). While the net effect of increased nutrients in mangrove forests is complex and not fully understood (Mack et al. 2024), these fertilization effects can come at the expense of seedling root growth (Gillis et al. 2019), soil microbial biodiversity (Craig et al. 2021), and potentially carbon storage (Mack et al. 2024).

Blue carbon ecosystems are increasingly included in mitigation strategies (Macreadie et al. 2017; Serrano et al. 2019), promoting restoration and conservation. These findings confirm their substantial capacity to capture carbon (Bouillon et al. 2008; Breithaupt et al. 2012; Breithaupt and Steinmuller 2022; Twilley et al. 1992) and highlight their resilience to tropical storms and hurricanes. If used in the global carbon market, the stability or impermanence of their carbon will be a notable concern, given the risk of tropical storms and other disturbances (Macreadie et al. 2021). Understanding that mangrove‐dominated ecosystems can recover lost carbon quickly may support their inclusion as nature‐based solutions for natural carbon capture, widely recognized as essential to avoiding dangerous climate change over the next two or three decades (Balmford et al. 2023).

### 4.3 Study Limitations

The differences between measured and modeled *R*_eco_ during recovery highlight the potential gap in scaling disturbance impacts. The response curve framework scales eddy covariance fluxes through modeled relationships between carbon exchange parameters and LAI. Under disturbance conditions, when LAI is reduced, these relationships are dynamic and are often not incorporated into other flux scaling efforts. It is well known that scaling fluxes through process‐based modeling leads to lower uncertainty at regional scale carbon budgets (Goetz et al. 2012; Liu et al. 2011), and here we show lower modeled *R*_eco_ rates compared to flux measurements at the fall forest site. While the light response curve methodology can account for disturbance effects, the model does not represent some carbon efflux. Few studies have examined net changes to ecosystem‐level carbon fluxes following hurricanes compared to other disturbances (Liu et al. 2011), and our study complements this work by elucidating land‐atmosphere CO_2_ exchange and related LAI recovery dynamics.

## 5 Conclusions

Recovery from hurricane disturbances to coastal ecosystems is complex and multifaceted. Using our long‐term record of eddy covariance data, we provide new estimates of hurricane recovery of coastal mangrove forests, showing that the high recovery debt incurred requires less than 4 years to recover all lost carbon. With hurricanes and tropical cyclones impacting approximately 45% of the total land area of mangrove forests, understanding the disturbance recovery timescale is a critical component of projecting the future of these ecosystems as the number of natural and anthropogenic stresses on coastal systems increases in the future under a warming climate.

## Author Contributions

**David Reed:** conceptualization, data curation, formal analysis, investigation, methodology, visualization, writing – original draft, writing – review and editing. **Selena Chavez:** formal analysis, visualization, writing – review and editing. **Edward Castañeda‐Moya:** data curation, funding acquisition, resources, writing – review and editing. **Steven F. Oberbauer:** funding acquisition, resources, writing – review and editing. **Tiffany Troxler:** data curation, funding acquisition, resources, writing – review and editing. **Sparkle Malone:** conceptualization, data curation, funding acquisition, methodology, resources, supervision, writing – original draft, writing – review and editing.

## 6 Conflicts of Interest

The authors declare no conflicts of interest.

## Supporting information


Data S1.

## Data Availability

The data and that support the findings of this study are openly available in Zenodo at https://doi.org/10.5281/zenodo.14947394. LAI data were obtained from the NASA Distributed Active Archive Center at http://doi.org/10.5067/MODIS/MYD15A2H.006. Eddy covariance data were obtained from AmeriFlux at https://doi.org/10.17190/AMF/1246105(US‐Skr) and https://doi.org/10.17190/AMF/2331383(US‐TaS).
