# Supplementary material for: Resilience to Hurricanes Is High in Mangrove Blue Carbon Forests
Source: Glob Chang Biol. 2025 Mar 19;31(3):e70124. doi: 10.1111/gcb.70124 (PMC11920944; doi:10.1111/gcb.70124)
Supplement: Supplementary file 1 — Data S1. [file GCB-31-e70124-s001.docx]

**Supporting Information for**

**Resilience to Hurricanes is High in Mangrove Blue Carbon Forests**

**Authorship list:**

David E. Reed, Selena Chavez, Edward Castañeda-Moya, Steve F. Oberbauer, Tiffany Troxler, Sparkle Malone

**Supporting Information Text**

**Data Aggregation Methods**

Eddy covariance fluxes were processed at 30-minute timescales following standard protocols. With eddy covariance data, there are typically discrete frequencies within the time series that carry most of the signal, notably the diel and seasonal timescales (Poe et al., 2020; Stoy et al., 2009). Since LAI data was collected at 8-day timescales, an analysis that includes diel frequency is impossible. To reduce noise in both datasets and gaps in the LAI dataset, both eddy covariance and LAI data were aggregated over 24-day periods. 24-day periods were chosen to minimize data loss in the LAI data at the end of each year. LAI data was aggregated using the maximum value of observations within each 24-day period. LAI data is synchronized to January 1^st^ of each year and 24-day aggregation leads to 5 days of lost data (6 on lead years), while a 32-day aggregation period (aggregation of four LAI samples) would result in 13 days of lost data (14 for leap years). For eddy covariance data, aggregation of flux rates were done by calculating the mean 30-minute flux rate of each half hour period over the course of a day (mean flux rate of 1:00-1:30 AM, 1:30-2:00 AM, ect), and then taking the mean of that composite diel time series. This was done to limit bias from missing nighttime data.Aggregation of parameters was done by fitting light response curves for 24-day days (Section 2.4). The curve fitting algorithm used stable parameter estimates, with less than 5% of parameters classified as outliers, particularly for the quantum yield and A_max_ parameters. For LAI data, aggregation is by taking the maximum value over the period (section 2.5).

To examine the effect of aggregation on results, data was aggregated into 8-day and 40-day periods. SI Figure 1 shows LAI data with 8-day, 24-day, and 40-day aggregation amounts. SI Figure 2 shows LAI and model results at the three aggregation levels. LAI means, and variance increased ~4% due to aggregation due to the maximum value being selected over each period, while eddy covariance light response curve parameters of respiration changed by ~1%, quantum yield by ~20%, and Amax by <1% (SI Figure 2). The nonlinear mixed effect model for light response curve parameters described in Eq. 4 (Section 2.6) was run on data aggregated at the three levels, with model results generally improving from 8-day to 24-day or 40-day periods (SI Figure 2i, SI Table 1-3).

**Text S2 Time Series Relative Non-Stationarity and Recovery Test Methods**

Adapted from the methods of Vickers and Mahrt (1997), we define relative non-stationarity in Eq. 5 in Section 2.7. We examined multiple time windows in which the relative non-stationarity test would be valid. Using air temperature as an annual periodical time series, relative non-stationarity was calculated over moving 1-year, 2-year, and 3-year windows (SI Figure 4) with little difference between window lengths. With little difference noted, a 3-year window was used for RNS calculations to diminish the effects of small gaps on the non-stationarity test. Due to the nature of the regression over moving 3-year windows, RNS calculations can detect non-stationarity in time series before they occur. In the case of LAI decrease, for the three preceding years, RNS is first increasing then decreases, as the regression window has the hurricane disturbance first at the end of the regression window, then middle, then start. This translates to the most negative RNS values being co-located at the hurricane landfall date, with RNS values trending back towards values of 1 as the regression windows move forward from the disturbance. Also, due to the 3-year window, RNS calculations end three years before the end of the data as seen on the difference in timescales in panels a and b in SI Figures 4-9.

Using the 95^th^, 90^th^, and 85^th^ percentiles of RNS values across the entire record as threshold values, the recovery dates can be determined as the final period under a given threshold. In some cases, the disturbance-recovery lagged from the hurricane's landfall, and interpretation is needed to determine the recovery timescale. For Reco and Amax at the Mangrove Forest site, the RNS is declining after the landfall of Irma, which we interpret as a decline due to disturbance. At the forest site, quantum yield has a potential disturbance recovery but has lagged in time since Hurricane Irma.

**Supporting Information Figures**

**Supporting Information Figure 1**


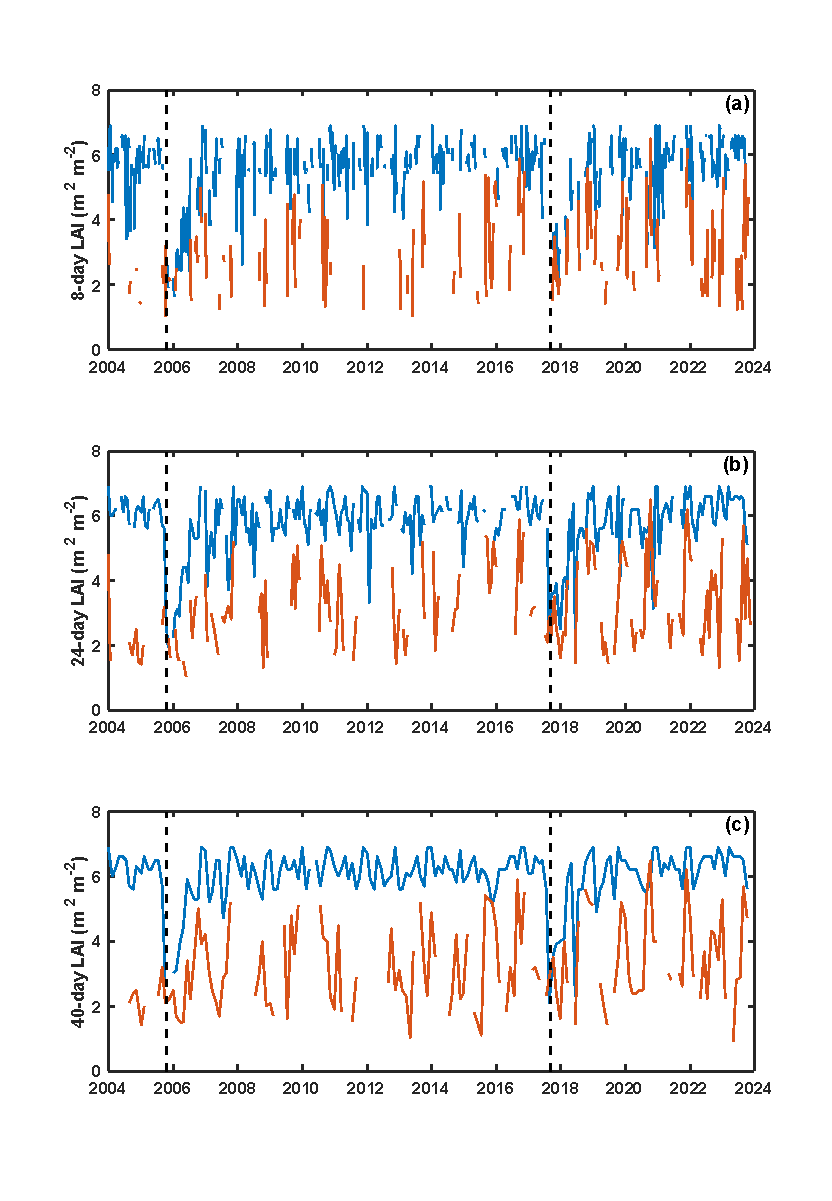


**Supporting Information Figure 1 Caption**

Time series of LAI at the mangrove forest (blue) and scrub (orange) sites shown at 8-day timescale (panel a), aggregated to 24-day timescale (panel b) and aggregated to 40-day timescale (panel c).

**Supporting Information Figure 2**


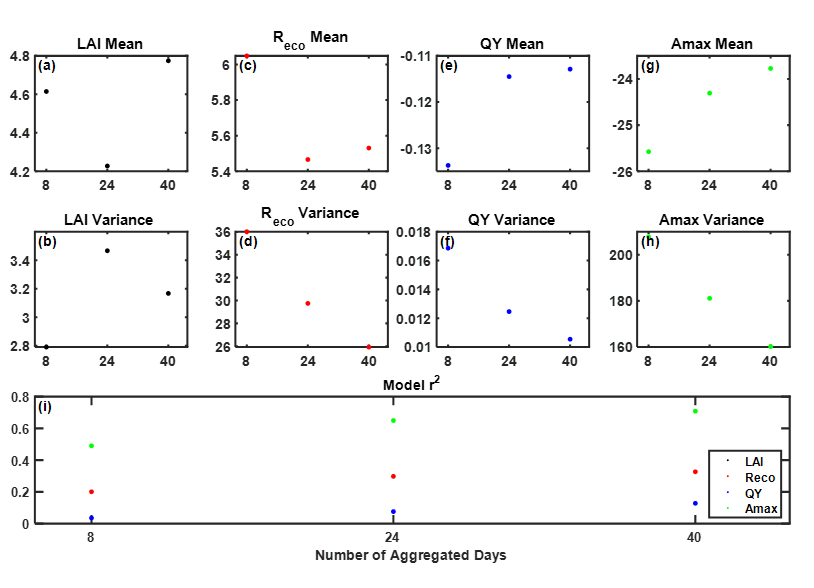


**Supporting Information Figure 2 Caption**

Summary statistics of carbon cycling model results based on 8-day, 24-day, and 40-day aggregation timescales, with LAI, ecosystem respiration (R_eco_), quantum yield (QY), and maximum assimilation of carbon A_max_ mean (panels a,c,e,g) and variances (panels b,d,f,h). Also shown is model r^2^ values for each aggregation timescale (panel i).

**Supporting Information Figure 3**


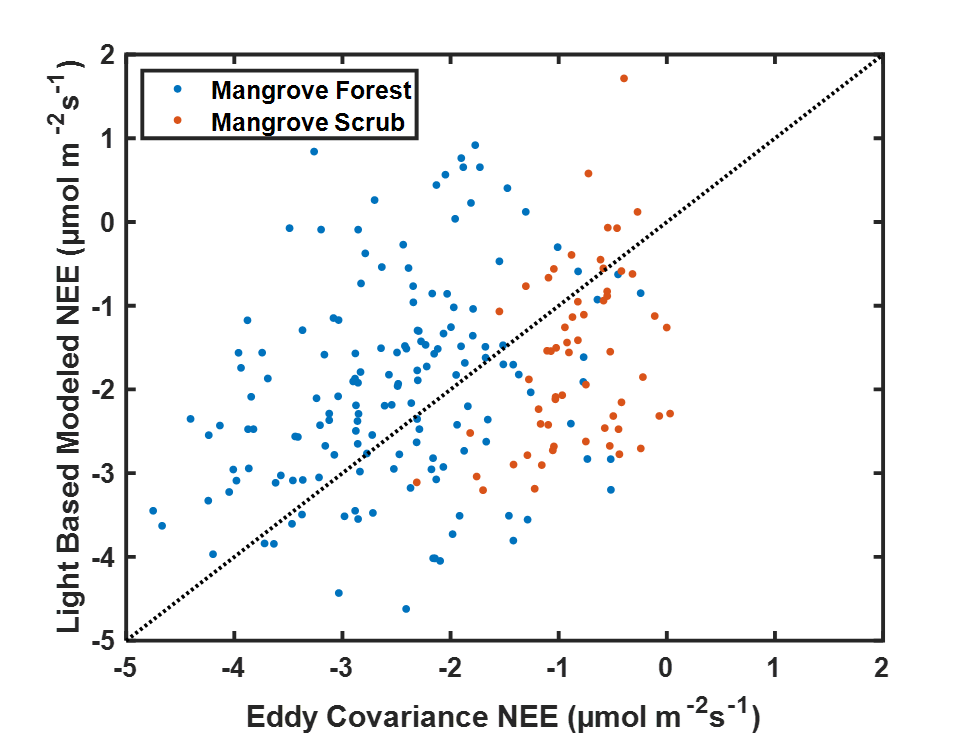


**Supporting Information Figure 3 Caption**

Comparison of mean half hour NEE rates from eddy covariance measurements and light-based modeled NEE from mangrove forest (blue) and scrub (orange) sites at 24-day averaged timescales.

**Supporting Information Figure 4**

**
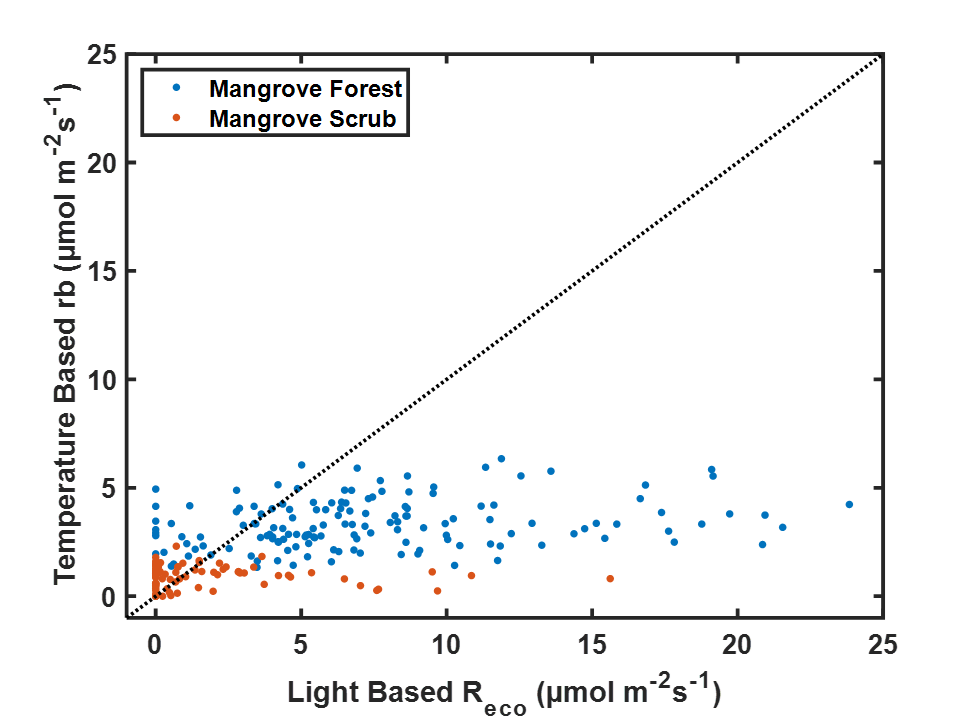
**

**Supporting Information Figure 4 Caption**

Comparison of light-based response curve respiration parameter (R_eco_) and temperature-based response curve base respiration parameter (rb) from mangrove forest (blue) and scrub (orange) at 24-day averaged timescales.

**Supporting Information Figure 5**

**
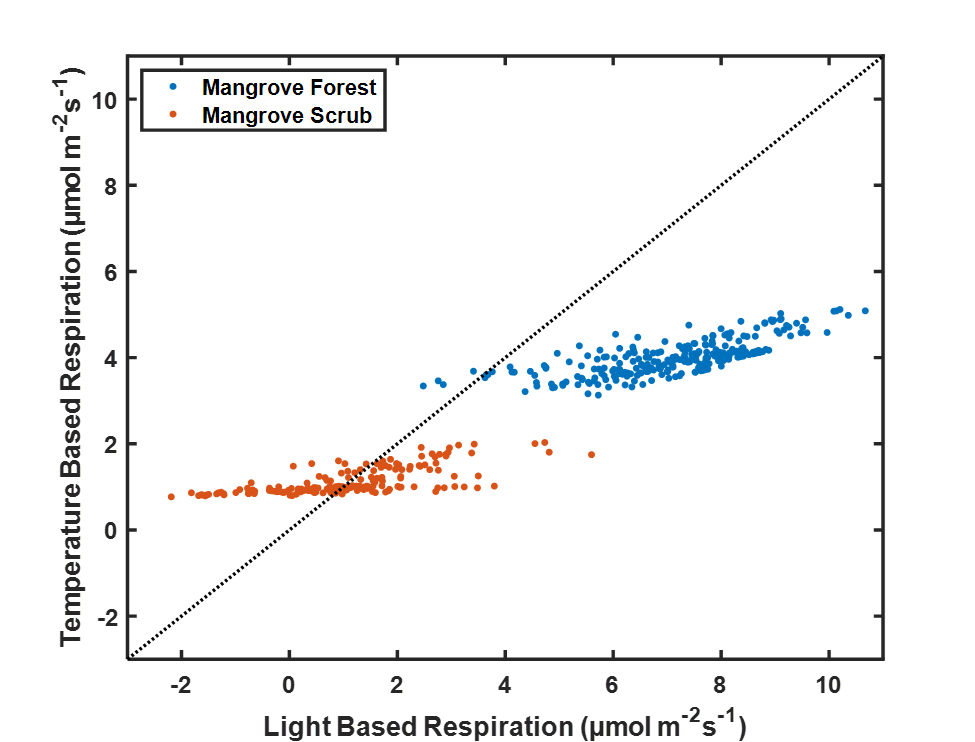
**

**Supporting Information Figure 5 Caption**

Comparison of light based response curve average respiration rate and temperature based response curve average respiration rate from mangrove forest (blue) and scrub (orange) at 24-day averaged timescales.

**Supporting Information Figure 6**


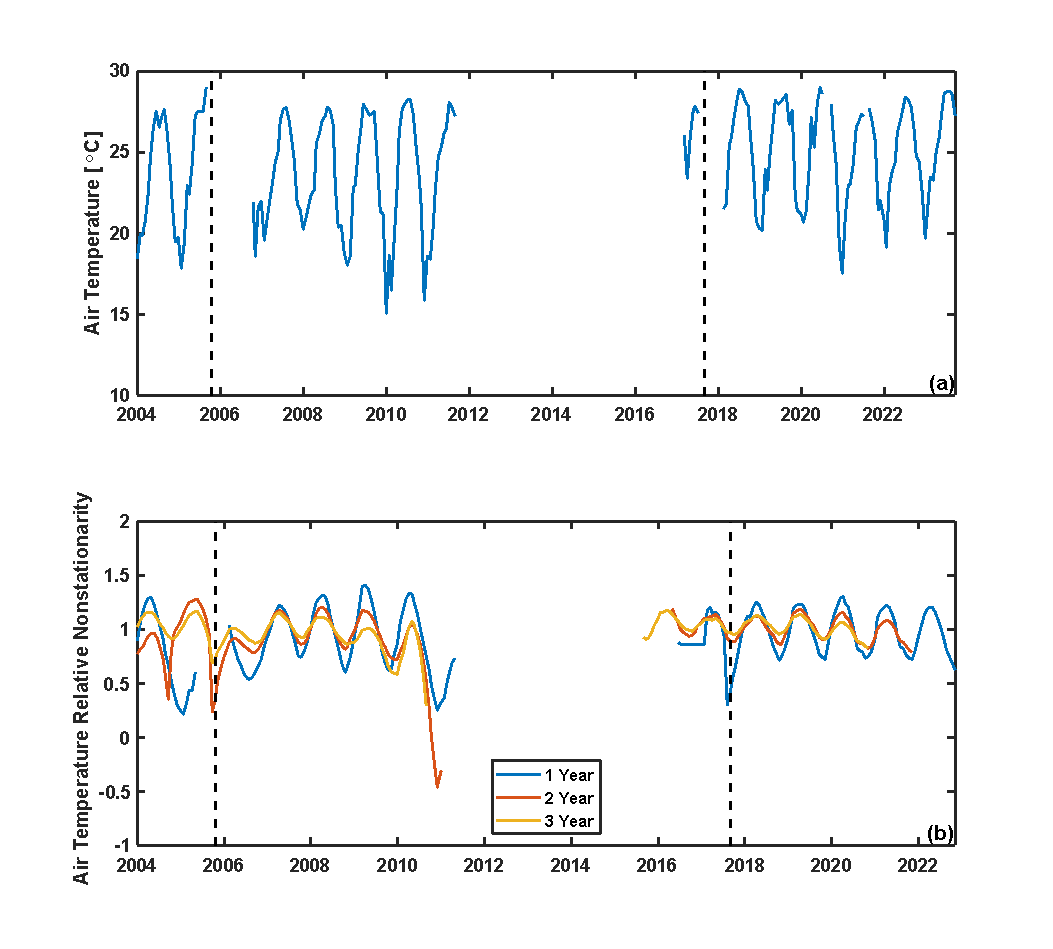


**Supporting Information Figure 6 Caption**

Time series of air temperature measurements from mangrove forest site (panel a) with relative non-stationary calculated over 1-year, 2-year, and 3-year periods (panel b). Landfall dates of Wilma (2005) and Irma (2017) are shown with a dashed line.

**Supporting Information Figure 7**


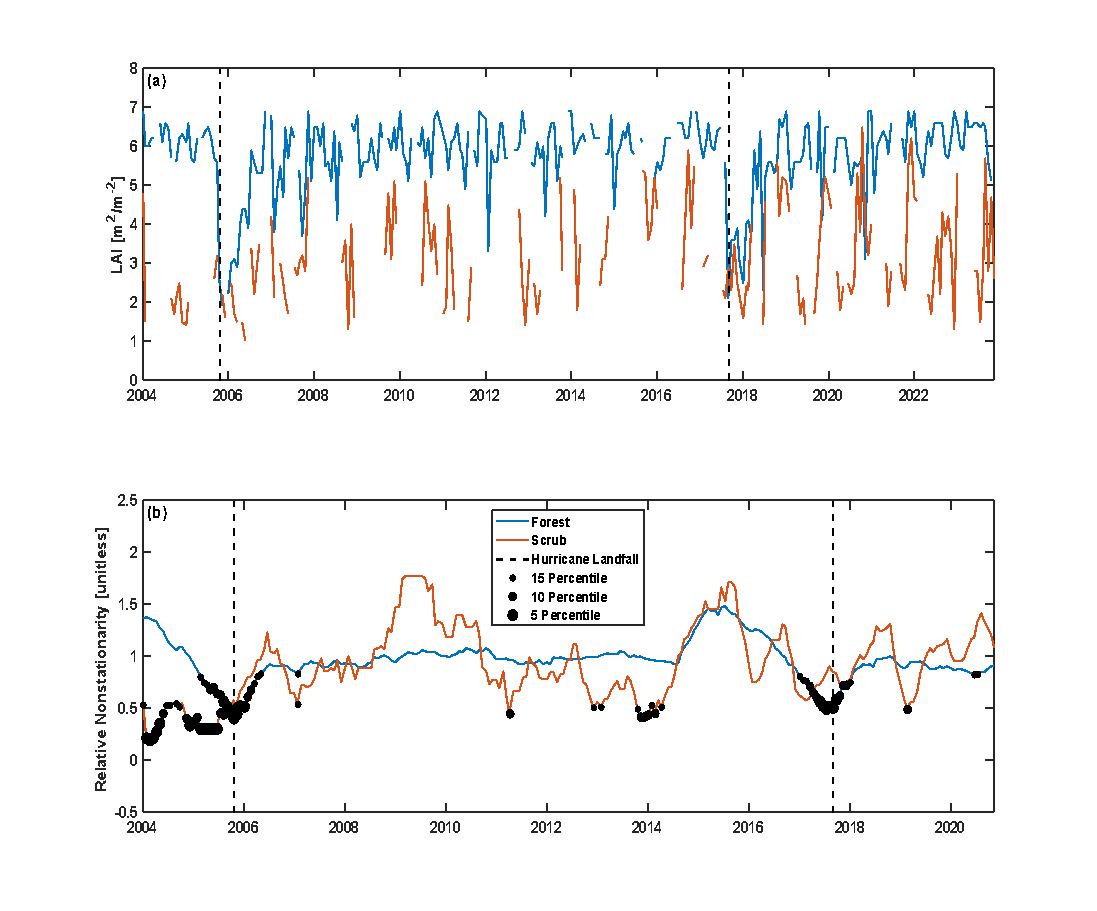


**Supporting Information Figure 7 Caption**

Time series of LAI from mangrove forest (blue) and scrub (orange) sites (panel a) with relative nonstationary LAI time series at both sites (panel b). Dates below the 5^th^, 10^th^, and 15^th^ percentile thresholds of each relative nonstationary time series are shown highlighted with black circles. Landfall dates of Wilma (2005) and Irma (2017) are shown with a dashed line.

**Supporting Information Figure 8**


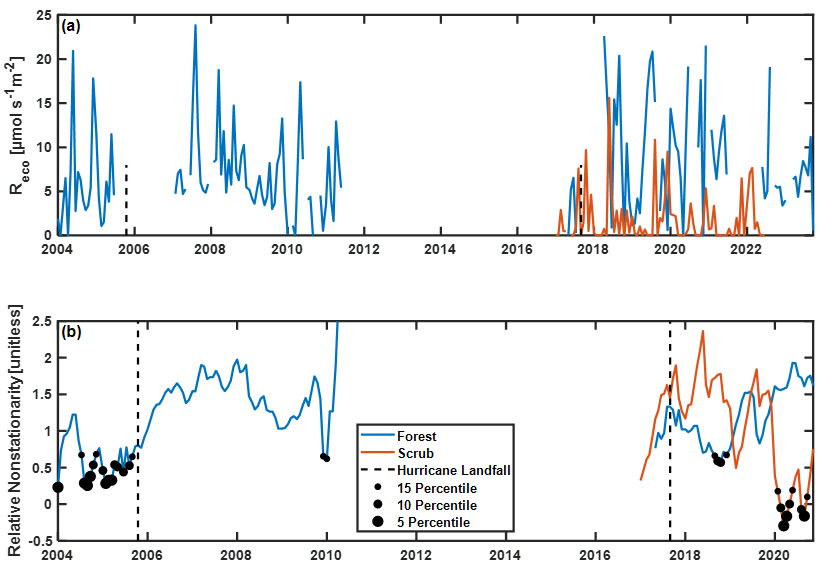


**Supporting Information Figure 8 Caption**

Time series of ecosystem respiration (R_eco_) from mangrove forest (blue) and scrub (orange) sites (panel a) with relative nonstationary ecosystem respiration time series at both sites (panel b). Dates below the 5^th^, 10^th^, and 15^th^ percentile thresholds of each relative nonstationary time series are shown highlighted with black circles. Landfall dates of Wilma (2005) and Irma (2017) are shown with a dashed line.

**Supporting Information Figure 9**


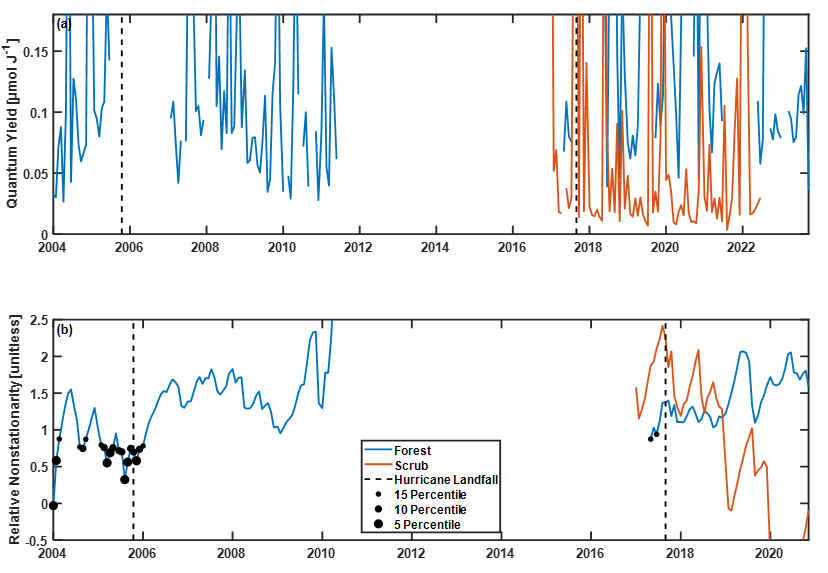


**Supporting Information Figure 9 Caption**

Time series of quantum yield from mangrove forest (blue) and scrub (orange) sites (panel a) with relative nonstationary QY time series at both sites (panel b). Dates below the 5^th^, 10^th^, and 15^th^ percentile thresholds of each relative nonstationary time series are shown highlighted with black circles. Landfall dates of Wilma (2005) and Irma (2017) are shown with a dashed line.

**Supporting Information Figure 10**


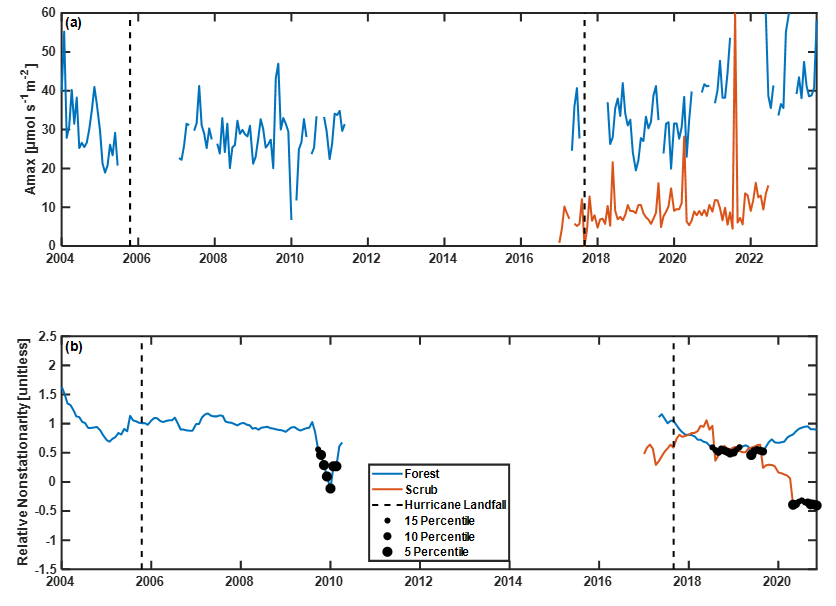


**Supporting Information Figure 10 Caption**

Time series of maximum photosynthesis (Amax) from mangrove forest (blue) and scrub (orange) sites (panel a) with relative nonstationary maximum photosynthesis time series at both sites (panel b). Dates below the 5^th^, 10^th^, and 15^th^ percentile thresholds of each relative nonstationary time series are shown highlighted with black circles. Landfall dates of Wilma (2005) and Irma (2017) are shown with a dashed line.

**Figure 11 Caption**


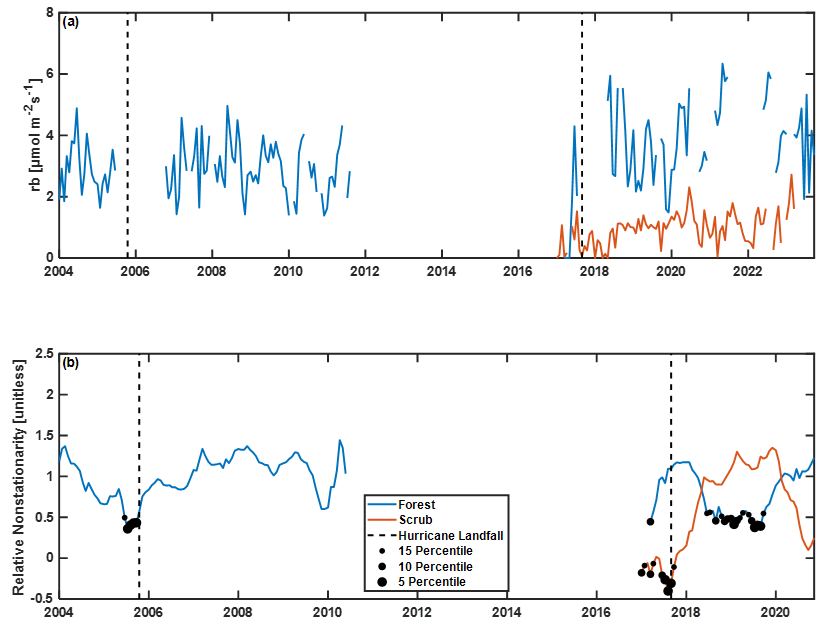


**Supporting Information Figure 11 Caption**

Time series of base respiration (rb) from mangrove forest (blue) and scrub (orange) sites (panel a) with relative nonstationary maximum photosynthesis time series at both sites (panel b). Dates below the 5^th^, 10^th^, and 15^th^ percentile thresholds of each relative nonstationary time series are shown highlighted with black circles. Landfall dates of Wilma (2005) and Irma (2017) are shown with a dashed line.

**Figure 12 Caption**

**
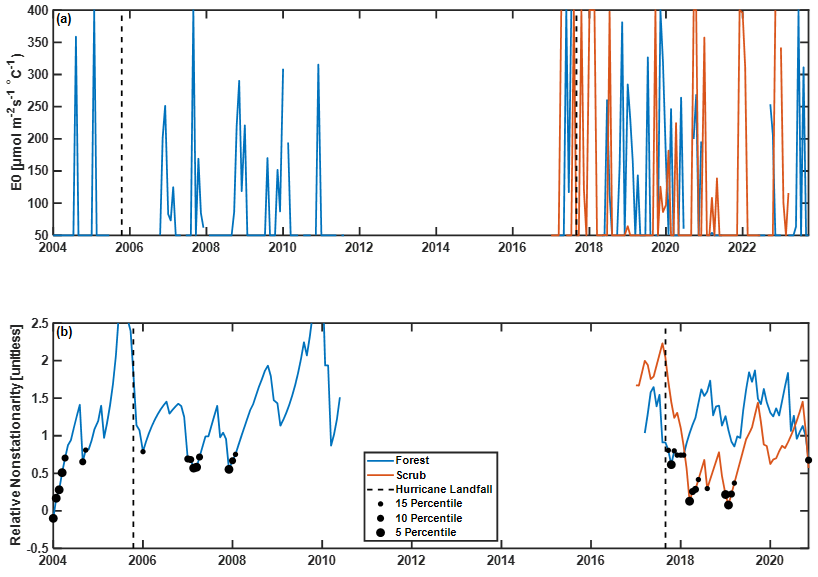
**

**Supporting Information Figure 12 Caption**

Time series of temperature sensitivity (E0) from mangrove forest (blue) and scrub (orange) sites (panel a) with relative nonstationary maximum photosynthesis time series at both sites (panel b). Dates below the 5^th^, 10^th^, and 15^th^ percentile thresholds of each relative nonstationary time series are shown highlighted with black circles. Landfall dates of Wilma (2005) and Irma (2017) are shown with a dashed line.

**Supporting Information Figure 13**

**
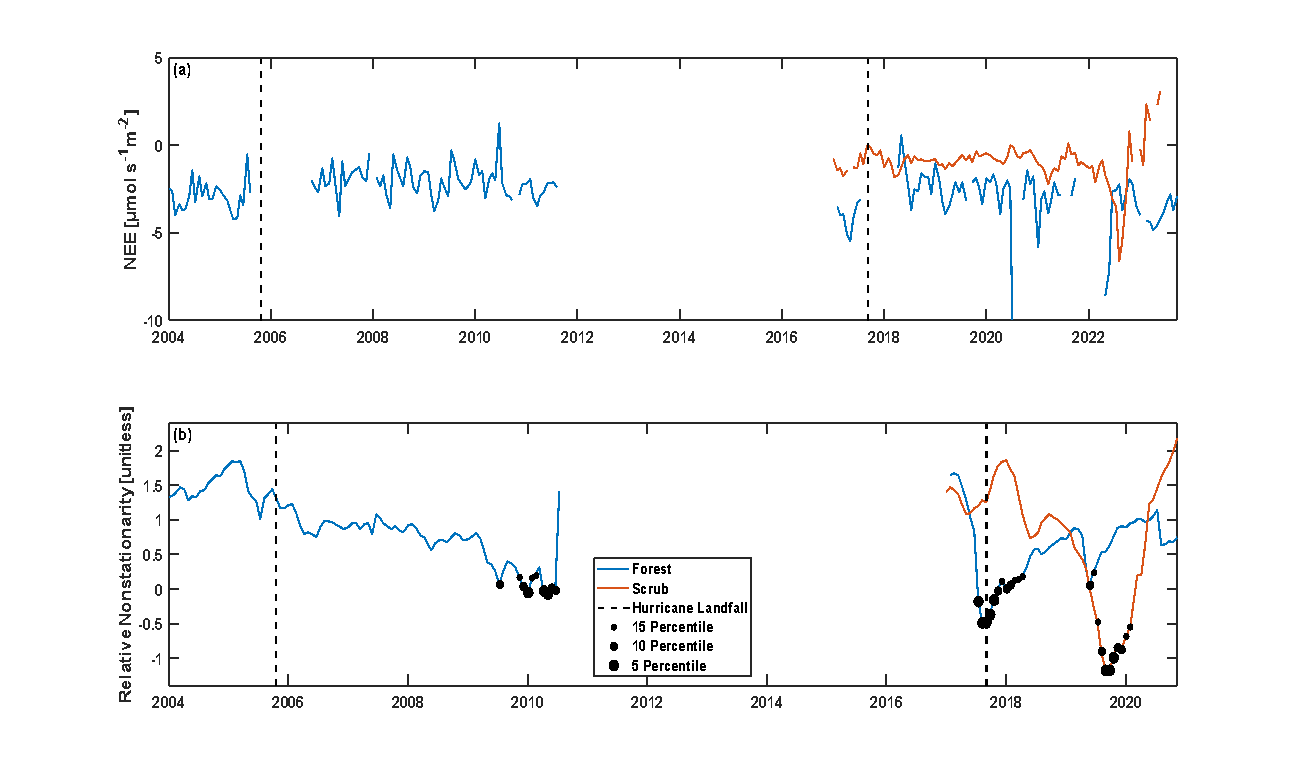
**

**Supporting Information Figure 13 Caption**

Time series of Net Ecosystem Exchange (NEE) from mangrove forest (blue) and scrub (orange) sites (panel a) with relative nonstationary NEE time series at both sites (panel b). Dates below the 5^th^, 10^th^, and 15^th^ percentile thresholds of each relative nonstationary time series are shown highlighted with black circles. Landfall dates of Wilma (2005) and Irma (2017) are shown with a dashed line.

**Supporting Information Figure 14**


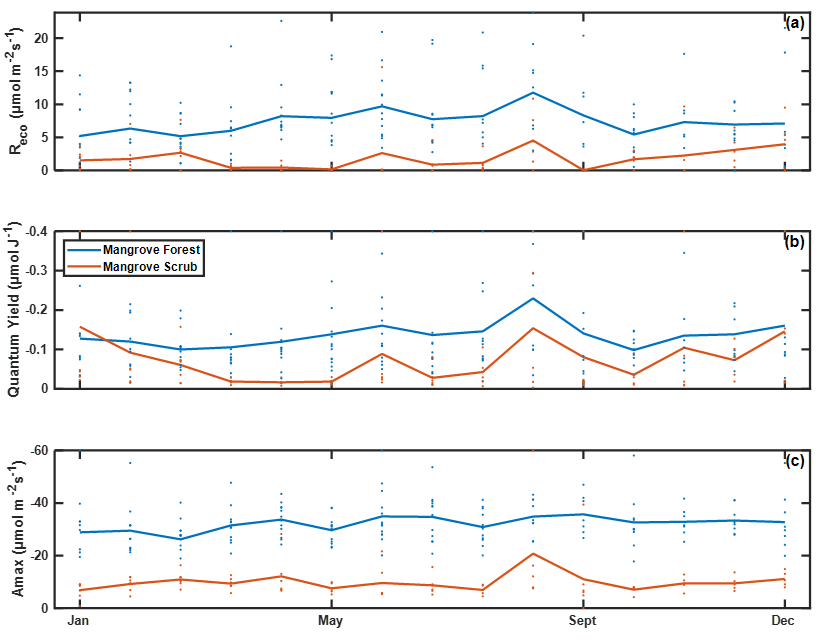


**Supporting Information Figure 14 Caption**

Annual average time series of ecosystem light-based respiration (R_eco_; panel a), quantum yield (panel b), and maximum photosynthesis (Amax; panel c) from mangrove forest (blue) and scrub (orange) sites.

**Supporting Information Table**

**Supporting Information Table 1**

| Site | Average Respiration Parameter Value [µmol s-1 m-2] | Average Modeled Respiration Rate [µmol s^-1^ m^-2^] |
| --- | --- | --- |
| Tall Forest | LRC R_eco_: 7.49  TRC rb: 3.22 | LRC: 7.14  TRC: 4.00 |
| Scrub Forest | LRC R_eco_: 1.76  TRC rb: 0.96 | LRC: 1.09  TRC: 1.12 |

**Supporting Information Table 1 Caption**

Mean respiration parameters from light response curves (LRC R_eco_) and temperature response curves (TRC rb) approaches and mean modeled respiration rates from light and temperature approaches at the mangrove forest and scrub sites. Parameters fit for 24-day periods, while modeled respiration rates are the mean of 24-day periods from the entire study period of 2004-2023.

**Supporting Information References**

Poe, J., Reed, D. E., Abraha, M., Chen, J., Dahlin, K. M., & Desai, A. R. (2020). Geospatial coherence of surface-atmosphere fluxes in the upper Great Lakes region. *Agricultural and Forest Meteorology, 295*, 108188.

Reichstein, M., Falge, E., Baldocchi, D., Papale, D., Aubinet, M., Berbigier, P., Bernhofer, C., Buchmann, N., Gilmanov, T., Granier, A., Grünwald, T., Havránková, K., Ilvesniemi, H., Janous, D., Knohl, A., Laurila, T., Lohila, A., Loustau, D., Matteucci, G., Meyers, T., Miglietta, F., Ourcival, J.-M., Pumpanen, J., Rambal, S., Rotenberg, E., Sanz, M., Tenhunen, J., Seufert, G., Vaccari, F., Vesala, T., Yakir, D. and Valentini, R. (2005), On the separation of net ecosystem exchange into assimilation and ecosystem respiration: review and improved algorithm. Global Change Biology, 11: 1424-1439. https://doi.org/10.1111/j.1365-2486.2005.001002.x

Stoy, P. C., Richardson, A. D., Baldocchi, D. D., Katul, G. G., Stanovick, J., Mahecha, M. D., et al. (2009). Biosphere-atmosphere exchange of CO 2 in relation to climate: a cross-biome analysis across multiple time scales. *Biogeosciences, 6*(10), 2297-2312.

Vickers, D., & Mahrt, L. (1997). Quality control and flux sampling problems for tower and aircraft data. *Journal of atmospheric and oceanic technology, 14*(3), 512-526.
